# Europium in plagioclase-hosted melt inclusions reveals mantle melting modulates oxygen fugacity

**DOI:** 10.1038/s41467-024-47224-5

**Published:** 2024-04-08

**Authors:** Nicholas Dygert, Gokce K. Ustunisik, Roger L. Nielsen

**Affiliations:** 1 Department of Earth, Environmental and Planetary Sciences, University of Tennessee, Knoxville, 1621 Cumberland Ave, 602 Strong Hall, Knoxville, TN 37996 USA; 2 Department of Geology and Geological Engineering, South Dakota School of Mines and Technology, 501 E. St. Joseph St., Rapid City, SD 57701 USA; 3 Department of Earth and Planetary Sciences, American Museum of Natural History, 200 Central Park West, New York, NY 10024 USA

**Keywords:** Geochemistry, Geodynamics, Petrology

## Abstract

To gain insights into the composition and heterogeneity of Earth’s interior, the partial pressure of oxygen (oxygen fugacity, or *f*O_2_) in igneous rocks is characterized. A surprising observation is that relative to reference buffers, *f*O_2_s of mantle melts (mid-ocean ridge basalts, or MORBs) and their presumed mantle sources (abyssal peridotites) differ. Globally, MORBs have near-uniform *f*O_2_s, whereas abyssal peridotites vary by about three orders of magnitude, suggesting these intimately related geologic reservoirs are out of equilibrium. Here, we characterize *f*O_2_s of mantle melting increments represented by plagioclase-hosted melt inclusions, which were entrapped as basaltic melts migrated from their sources toward the seafloor. At temperatures and *f*O_2_s constrained by rare earth element distributions, a range of *f*O_2_s consistent with the abyssal peridotites is recovered. The *f*O_2_s are correlated with geochemical proxies for mantle melting, suggesting partial melting of Earth’s mantle decreases its *f*O_2_, and that the uniformity of MORB *f*O_2_s is a consequence of the melting process and plate tectonic cycling.

## Abstract (web summary)

Distributions of the multivalent element Europium are used to recover the partial pressure of oxygen from basaltic mantle melt inclusions trapped in plagioclase crystals, suggesting Earth’s mantle is reduced by partial melting.

## Introduction

Application of several oxybarometric methods to estimate the partial pressure of oxygen (oxygen fugacity, or *f*O_2_) from primitive and glassy mid-ocean ridge basalts (MORBs) recovers *f*O_2_s approximating the fayalite-magnetite-quartz (FMQ) buffer, independent of sampling locality^1–4^. In contrast, investigations of abyssal peridotites drilled and dredged from amagmatic exposures along ridges and transform faults recover *f*O_2_s varying by ~three orders of magnitude about the FMQ buffer^5–7^. Abyssal peridotites originate from Earth’s convecting upper mantle and are representative of the MORB source, from which basalts are derived by channelized, near-fractional adiabatic decompression melting^8^. Some scatter among oxybarometric *f*O_2_ determinations for MORBs and abyssal peridotites may reflect analytical or method bias, but among the oxybarometers, broad agreement in the order of magnitude ranges of *f*O_2_s recorded by peridotites and basalts, and the proximity of averaged *f*O_2_ values to the FMQ buffer suggests that the oxybarometers are fairly accurate. Thus, taken at face value, the inconsistency in *f*O_2_ between abyssal peridotites and oceanic basalts suggests that heterogeneity in *f*O_2_ is a characteristic of the MORB source, but that on average, the MORB source approximates the FMQ buffer^9^, and/or that *f*O_2_s recorded by peridotites are variably affected by different degrees of partial melting and melt-rock reaction (metasomatism or melt impregnation). Until recently, these suppositions were challenging to test, as characterization of the *f*O_2_ of near-fractional MORB melts trapped in mineral inclusions was fraught with complications including open system behavior owing to rapid exchange kinetics^10,11^, and/or analytical difficulties (e.g., the challenge of doubly polishing a melt inclusion for X-ray spectroscopic analysis).

Here we apply an Eu-in-plagioclase-melt oxybarometer^12^ to recover *f*O_2_s from melt inclusions in plagioclase phenocrysts extracted from MORBs. The technique uses Eu distributions between melt inclusions and their plagioclase hosts, which can be measured in-situ, to determine *f*O_2_s. We additionally apply a rare earth element (REE)-in-plagioclase-melt thermometer to the samples to recover trivalent REE temperatures, which are critical for calculating *f*O_2_s and appropriately referencing the *f*O_2_s to a buffer at the melt inclusion entrapment conditions. From the melt inclusion data, application of the Eu-in-plagioclase-melt oxybarometer and REE in plagioclase-melt thermometer recovers a ~3 order of magnitude *f*O_2_ range relative to the FMQ buffer, in agreement with determinations from abyssal peridotites. The recovered *f*O_2_s are correlated with geochemical indices of fractionation, suggesting the *f*O_2_ of Earth’s mantle is modulated by partial melting. Application of the Eu-based oxybarometer to plagioclase phenocryst-host glass pairs in an additional subset of MORB samples previously characterized by X-ray absorption near edge spectroscopy (XANES)^3^ recovers the earlier determinations, supporting the accuracy of the new Eu-in-plagioclase-melt-based *f*O_2_s. Uncertainty analysis suggests the newly determined *f*O_2_s are accurate within (on average) 0.57 log units.

## Results and discussion

### An Eu-in-plagioclase-melt oxybarometer

Eu is a multivalent element exhibiting divalent character under reducing conditions and trivalent character under oxidizing conditions in geologic systems. Because divalent Eu has an ionic radius similar to Ca, Eu readily substitutes into the Ca site in plagioclase structure under reducing conditions, while under oxidizing conditions it is moderately to highly incompatible, depending on plagioclase anorthite content^12,13^. If the partitioning behavior of the divalent and trivalent Eu species can be accurately predicted, magmatic oxygen fugacity (*f*O_2_) recorded by quenched samples can be determined from the ratio of measured Eu concentrations in plagioclase-glass pairs^14^ (i.e., the Eu partition coefficient, ${D}_{{Eu}}$), assuming the glass is representative of melt in equilibrium with the coexisting plagioclase crystal just before quenching. This oxybarometric model approach^12,14,15^ is described below.

The plagioclase-melt partition coefficient for Eu is a combination of contributions from the divalent and trivalent Eu species in the plagioclase and coexisting melt,

1 $${D}_{{Eu}}=\frac{{\left[{M}_{{{Eu}}^{2+}}\right]}_{{plag}}+{\left[{M}_{{{Eu}}^{3+}}\right]}_{{plag}}}{{\left[{M}_{{{Eu}}^{2+}}\right]}_{{melt}}+{\left[{M}_{{{Eu}}^{3+}}\right]}_{{melt}}} .$$

where brackets indicate mass fractions. The proportions of divalent and trivalent Eu in in the silicate melt can be described as a function of *f*O_2_ for a reduction reaction,

2 $${{Eu}}^{3+}{O}_{1.5}={{Eu}}^{2+}O+\frac{1}{4}{O}_{2} .$$

Defining an equilibrium constant for the reaction ($K$), we describe the activities of the reduced and oxidized species as products of their mole fractions ($x$) and activity coefficients ($\gamma$),

3 $$K=\frac{{{\gamma }_{{{Eu}}^{2+}O}\cdot x}_{{{Eu}}^{2+}O}\cdot {\left(f{O}_{2}\right)}^{\frac{1}{4}}}{{\gamma }_{{{Eu}}^{3+}{O}_{1.5}}\cdot {x}_{{{Eu}}^{3+}{O}_{1.5}}} .$$

Making the simplifying assumption that the activity coefficients for the reduced and oxidized Eu species in the melt are equal^15^,

4 $$K=\frac{{x}_{{{Eu}}^{2+}O}\cdot {\left(f{O}_{2}\right)}^{\frac{1}{4}}}{{x}_{{{Eu}}^{3+}{O}_{1.5}}} .$$

Converting mole fractions to mass fractions, the conversion factors in the numerator and denominator cancel, and

5 $$K=\frac{{\left[{M}_{{{Eu}}^{2+}}\right]}_{{melt}}\cdot {\left(f{O}_{2}\right)}^{\frac{1}{4}}}{{\left[{M}_{{{Eu}}^{3+}}\right]}_{{melt}}} .$$

Rearranging and substituting Eq. 5 into Eq. 1, we obtain an expression to model ${D}_{{Eu}}$ as a function of divalent and trivalent Eu partition coefficients (${D}_{{{Eu}}^{2+}}$ and ${D}_{{{Eu}}^{3+}}$), which must be predicted as a function of plagioclase composition, *f*O_2_ and the equilibrium constant $K$^14,15^,

6 $${D}_{{Eu}}=\frac{K\cdot {D}_{{{Eu}}^{2+}}+{D}_{{{Eu}}^{3+}}\cdot {\left(f{O}_{2}\right)}^{\frac{1}{4}}}{K+{\left(f{O}_{2}\right)}^{\frac{1}{4}}}.$$

Equation 6 can then be rearranged as an Eu-in-plagioclase melt oxybarometer,

7 $$\log \left({{fO}}_{2}\right)=-4\times \log \left(\frac{{D}_{{Eu}}^{3+}-{D}_{{Eu}}}{K\left({D}_{{Eu}}-{D}_{{Eu}}^{2+}\right)}\right) .$$

The divalent and trivalent Eu partition coefficients are calculated using the lattice strain model^16^,

8 $${D}_{i}={D}_{0}\exp \left(\frac{-4\pi E{N}_{A}}{{RT}}\left(\frac{{r}_{0}}{2}{\left({r}_{i}-{r}_{0}\right)}^{2}+\frac{1}{3}{\left({r}_{i}-{r}_{0}\right)}^{3}\right)\right),$$

where ${N}_{A}$ is Avogadro’s number, $E$ is the apparent Young’s modulus, *R* is the gas constant, ${r}_{0}$ is the ideal ionic radius of the lattice site, ${r}_{i}$ is the ionic radius of the substituting element (for the plagioclase ring site), in VIII-fold coordination^17^, and $T$ is the temperature in Kelvin. Partition coefficients for the divalent and trivalent Eu species (${D}_{{{Eu}}^{2+}}$ and ${D}_{{{Eu}}^{3+}}$) are predicted using models developed by Sun et al.^13^. The lattice strain model terms used in the trivalent Eu prediction are calculated using the following expressions^13^,

9 $${{{{{\mathrm{ln}}}}}}\left({D}_{0}^{3+}\right)=16.05-\frac{19.45+1.17{P}^{2}}{{RT}}\times {10}^{4}-5.17{({X}_{{Ca}})}^{2},$$

10 $${r}_{0}^{3+}=1.179,$$

11 $${E}^{3+}=196,$$

where $P$ is in GPa, ${X}_{{Ca}}$ is the number of Ca cations in the plagioclase formula calculated from measured data on an eight oxygen basis, ${r}_{0}^{3+}$ is in Å, and ${E}^{3+}$ is in GPa.

The lattice strain model terms used in the divalent Eu prediction are calculated using the following expressions, again developed by Sun et al.^13^,

12 $${{{{{\mathrm{ln}}}}}}\left({D}_{0}^{2+}\right)=\frac{6910-2542{P}^{2}}{{RT}}+2.39{({X}_{{Na}})}^{2},$$

13 $${r}_{0}^{2+}=1.189+0.075{X}_{{Na}},$$

14 $${E}^{2+}=719-487{r}_{0}^{2+},$$

where ${X}_{{Na}}$ is the number of cations in the plagioclase formula calculated on an eight oxygen basis, $P$ is in GPa, ${r}_{0}^{2+}$ is in Å, and ${E}^{2+}$ is in GPa. The ${r}_{0}$ and $E$ terms (Eqs. 10, 11, 13 and 14) must be converted into meters and Pascals (respectively) before applying in Eq. 8. The relationships described by Eqs. 1–14 have been known for years to decades. In the current study, our contributions are integration of the aforementioned models, determination of the equilibrium constant $K$ using MORB-relevant experimental observations (see below), recasting the $T$-sensitive plagioclase-melt REE partitioning model^13^ into a mineral-melt thermometer (see Eqs. 16–19), application of Eqs. 1–19 to a new dataset, uncertainty analysis, and interpretation of the results.

The equilibrium constant $K$ was determined by nonlinear least squares regression from a dataset of plagioclase-melt partitioning experiments conducted under controlled *f*O_2_s (see Methods and Supplementary Figs. S1, S2 and S3 for details). The divalent and trivalent Eu partition coefficients are calculated for each experiment using Eqs. 8–14 according to the plagioclase compositions and experimental $P-T$ conditions. Because mid-ocean ridge basalts are the focus of the present study, we calculated $K$ using experiments characterizing terrestrial basaltic systems only (see Methods and Supplementary Fig. S1a).

15 $$K=9.13\times {10}^{-4}\pm 1.12\times {10}^{-4},$$

well within error of the value determined using a larger calibration dataset including simple, evolved, and lunar and Mars-relevant systems^12^. Uncertainty in the equilibrium constant (1σ) is calculated from the model fit residuals assuming asymptotic normal distribution about the parameter estimate (Supplementary Fig. S1b).

A thorough attempt was made to parameterize $K$ as a function of temperature and/or compositional terms (e.g., Schreiber^18^), but these additions did not significantly improve the quality of the fit. The calibrating data are well described by Eq. 6 using a constant $K$ regardless of $P-T$ conditions and melt composition (but note the $P-T$ and plagioclase composition dependence of the ${D}_{{{Eu}}^{2+}}$ and ${D}_{{{Eu}}^{3+}}$ predictions). Applying this constant $K$ value, Eq. 7 accurately recovers *f*O_2_s over 13 orders of magnitude of experimentally imposed *f*O_2_ variation (Supplementary Fig. S2) across a temperature range of 1127–1350 °C, except at high *f*O_2_s that are not geologically relevant, where ${D}_{{Eu}}$ is less responsive to *f*O_2_ variation (see Supplementary Fig. S3 for a demonstration). As it is based upon elemental partitioning of Eu between a basaltic melt inclusion and its host plagioclase, which is relatively insensitive to diffusive perturbation for quenched samples (e.g., MORBs), determination of *f*O_2_s by multivalent element partition coefficients may be less sensitive to degassing^19,20^, assimilation^2^, or charge transfer reactions upon quenching (e.g., exchange of an electron between trivalent Fe and divalent Cr, producing a glass with less trivalent Fe than its parent melt^21^) compared to techniques that determine *f*O_2_ as a proxy of the valence of redox sensitive elements in basaltic glasses^22^.

### A REE in plagioclase-melt thermometer

For partitioning of Eu between plagioclase and coexisting melt to be a representative proxy for *f*O_2_ at magmatic conditions, equilibration of the divalent and trivalent Eu species between the phases, and closure of the equilibrated plagioclase-melt system are required. As a means for evaluating trivalent REE equilibration conditions in the samples investigated here, we rearranged the trivalent element plagioclase-melt partitioning model of Sun et al.^13^ as a thermometer. Following the form of Liang et al.^23^ and Sun and Liang^24^,

16 $$T\left[{{{{{\mathrm{ln}}}}}}\left({D}_{i}\right)-A\right]+C=B,$$

where *T* is temperature in Kelvin, ${D}_{i}$ is the trivalent element of interest, $A$ is a function of plagioclase composition, $C$ is a function of pressure, and $B$ is a function of the properties of the plagioclase ring site and the radius of the substituting element. Rearranging Eq. 9 (Sun et al.^13^) and Eq. 8 (Wood and Blundy^16^) into the form of Eq. 16,

17 $$A=16.05-5.17{\left({X}_{{Ca}}\right)}^{2},$$

where ${X}_{{Ca}}$ is Ca in plagioclase in formula units, on an eight oxygen basis.

18 $$C=\frac{{10}^{4}}{R}\left(19.45+1.17{P}^{2}\right),$$

where *P* is pressure in GPa and *R* is the gas constant.

19 $$B=-\frac{4\pi E{N}_{A}}{R}\left(\frac{{r}_{0}}{2}{\left({r}_{0}-{r}_{i}\right)}^{2}-\frac{1}{3}{\left({r}_{0}-{r}_{i}\right)}^{3}\right),$$

where *E* is the Young’s modulus of lattice site (in Pascals), ${N}_{A}$ is Avagadro’s number, ${r}_{0}$ is the ideal ionic radius of the lattice site, and ${r}_{i}$ is the ionic radius of the substituting element (in meters). *E* and *r*_*0*_ are 196 GPa and 1.179 Å, respectively, for trivalent elements substituting into the ring site in plagioclase in eightfold coordination (Eqs. 10 and 11, Sun et al.^13^). Temperatures inverted from trace element partitioning experiments are generally in agreement with the imposed conditions and are shown in Supplementary Fig. S4.

To calculate temperatures from measured REE distributions, data are plotted in inversion diagrams (Supplementary Figs. S5–S7) and a line is regressed through the observations using a robust least squares fitting algorithm that excludes outliers (e.g., Eu, which falls off the trends defined by exclusively trivalent cations). The slope of the line corresponds to temperature, the y intercept is fixed by an assumed pressure (0.4 GPa for all samples considered here^25–27^). The measured data ideally exhibit linear behavior in the temperature inversion space; in cases where REEs are fractionated relative to an equilibrium distribution the data are rotated in the inversion diagram. Thus, application of this method provides a means to evaluate data quality beyond scrutiny of REE patterns, which should exhibit smooth and systematic variations when plotted in a chondrite-normalized spider diagram. Applying Eqs. 16–19 to REE and Y distributions between plagioclase and coexisting glasses recovers magmatic temperatures from 40 of 53 samples investigated in this study (Supplementary Figs. S5–S7). Assuming a pressure of 0.2 GPa reduces the recovered temperatures by 10 °C. Temperature uncertainties were estimated using uncertainties in the slope of the temperature inversions.

### Application of the Eu-based oxybarometer

We apply the Eu-in-plagioclase oxybarometer and trivalent REE in plagioclase-melt thermometer (Eqs. 7–19) to samples from the Juan de Fuca ridge, Blanco Fracture Zone, Gorda ridge, mid-Atlantic ridge, Southeast and Southwest Indian ridges (SEIR and SWIR, respectively), and Gakkel ridge. Eighteen glass-melt pairs reflect new data produced in this study from samples originally investigated by Lewis et al.^28^ (Juan de Fuca and Blanco), and Cottrell and Kelley^3^ (mid-Atlantic samples, whose iron oxidation states were previously constrained by Fe speciation analysis, by XANES). Major elements were analyzed at the University of Tennessee and Oregon State University, and trace elements were analyzed at the University of Texas at Austin. The new data are summarized in Supplementary Data 1 and 2. Additional compositional data were compiled from the literature^29–31^ and are summarized in Supplementary Data 3.

Four different sample types are investigated here: experimentally homogenized inclusion-host pairs (plotted as diamonds, this study; Nielsen et al.^29^), inclusion-host pairs that were not experimentally homogenized (plotted as triangles^30^, we note their glasses have MgO contents of 7.0–9.2 wt%), fractionation corrected inclusions (plotted as circles^31^), and plagioclase phenocryst-host glass pairs (plotted as squares, this study; Cottrell and Kelley^3^).

**Fig. 1:**
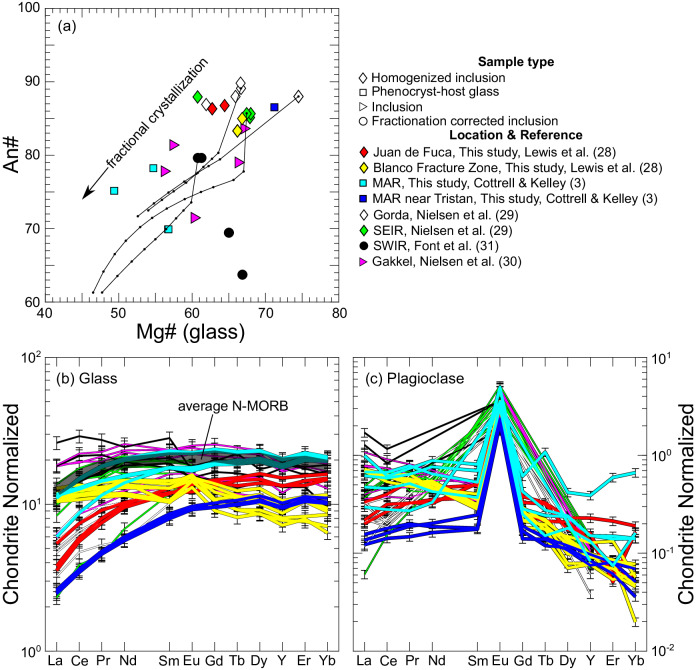
Compositions of samples investigated in this study. Plagioclase An# (100 × Ca/(Ca + Na + K), in moles) vs. coexisting glass Mg# (100 × Mg/(Mg + Fe^2+^+ Fe^3+^), in moles) (**a**); sample type is indicated by symbol type, color indicates locality, see legend. Thin black lines show models of fractional crystallization for four glass compositions representative of basaltic melts, black dots correspond to 5% increments (fractionating phase proportions and An#s were calculated using alphaMELTS; see Melting and fractionation models for details). Chondrite normalized REE abundances in glass and coexisting plagioclase are shown in (**b**) and (**c**) respectively; colors correspond to locality as indicated in the legend; thicker lines are new data produced in this study; thinner lines are data compiled from the literature. For comparison, global average “normal” mid-ocean ridge basalt (N-MORB)^33^ is shown as the thickest black semi-transparent line in (**b**). Iron species proportions are calculated using the model of Kress and Carmichael^47^ using *f*O_2_s determined from measured Eu distributions at the REE temperatures (Eqs. 7–19).

Glasses range from highly primitive to evolved, with Mg#s of 74.5–49 (where Mg# = 100 × Mg/(Mg + Fe^2+^+ Fe^3+^), in moles); coexisting plagioclase covary in primitive to refractory samples, with An#s of 90.9 to 63.7 (where An# = 100 × Ca/(Ca + Na + K), in moles) (Fig. 1a). The trend can be interpreted as reflecting variable depletion of a melt source (the depleted MORB mantle, or DMM^32^), with the highest Mg#s representing primitive, high degree melts, and low Mg# and An#s reflecting lower degree melts, and basalts that experienced significant crystal fractionation, as removal of olivine and plagioclase ± clinopyroxene extracts compatible Mg and normative anorthite from initially MORB-like melts. The measured glasses have smooth and systematic MORB-like REE patterns and are generally more depleted than the global average N-MORB^33^ (“normal” MORB), exhibiting variably positive or negative Eu anomalies (i.e., Eu abundances exceeding or less than the value interpolated from neighboring elements on a chondrite normalized REE diagram, Fig. 1b). Plagioclase exhibit steeply dipping light to heavy REE patterns (in accordance with REE compatibility in the ring site, which favors larger light REEs^12^), and universally positive Eu anomalies, though the Eu anomaly magnitudes vary among samples (Fig. 1c). Partition coefficients calculated using the measured REE concentrations are shown in Supplementary Fig. S8.

**Fig. 2:**
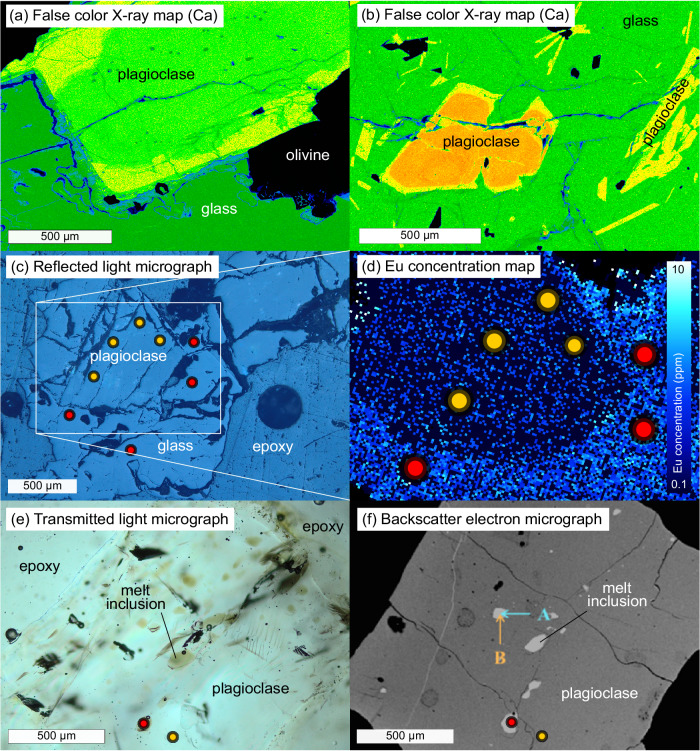
Representative images illustrating our sample characterization campaign. Samples with plagioclase-phenocryst pairs are shown in (**a**)–(**d**); a plagioclase crystal with melt inclusions is shown in (**e**) and (**f**). False color X-ray maps demonstrate anorthite content variations in plagioclase crystals that are mantled by rims that may be in equilibrium with host melt (**b**). Note lack of zoning in smaller plagioclase crystals in (**b**). The plagioclase crystal and host glass shown in (**c**) was mapped by LA-ICP-MS to characterize potential Eu heterogeneity; both phases are uniform within analytical uncertainty (**d**). The scale on the Eu concentration color bar is logarithmic. Filled circles in (**c**)–(**f**) show placement of LA-ICP-MS spots (not to scale); red indicates glass and yellow indicates plagioclase. In (**e**), a plagioclase crystal containing melt inclusions is mounted in epoxy and photographed in transmitted light, enabling observation of inclusions in the crystal deeper than the focal plane. Arrows in (**f**) show placement of electron microprobe traverses that demonstrate lack of zoning around plagioclase melt inclusions^29^ (Supplementary Data 4).

Application of Eqs. 7–19 requires major and trace element equilibrium between the coexisting glass and plagioclase crystal. We optically examined plagioclase and coexisting glass to check for quench modification, disequilibrium crystal morphologies or evidence of alteration, and only targeted fresh non-skeletal crystals and fresh glasses for analysis. We analyzed both crystal rims and cores to evaluate potential intrasample heterogeneity, and additionally collected X-ray and Eu maps of selected samples. Representative examples of the latter are shown in Fig. 2a, b, and d. Some phenocryst-host glass samples contained zoned plagioclase, and/or reaction textures that were avoided during trace element analysis; others are in apparent equilibrium with surrounding glasses (smaller grains in Fig. 2b). Backscattered electron micrographs and major and trace element analyses suggest that the phenocryst-melt inclusion samples are compositionally homogeneous, particularly over length scales relevant to chemical exchange between melt inclusions and their host crystals (Fig. 2f; also see Supplementary Data 4 and Nielsen et al.^29^). Eu mapping (conducted by laser ablation inductively coupled plasma mass spectrometry, LA-ICP-MS) did not reveal analytically resolvable intraphase heterogeneity beyond analytical uncertainty in the phenocryst-host glass or phenocryst-melt inclusion samples (e.g., Fig. 2d). Equipped with these carefully collected data, we proceeded to calculate *f*O_2_s, and trivalent REE temperatures for every sample recorded by measured trivalent glass-plagioclase REE distributions in our samples, and using additional data compiled from the literature. The REE temperatures are used to calculate *f*O_2_s, and to reference recovered *f*O_2_s against metal and mineral reaction buffers at conditions most relevant to the samples.

### Oxygen fugacities

**Fig. 3:**
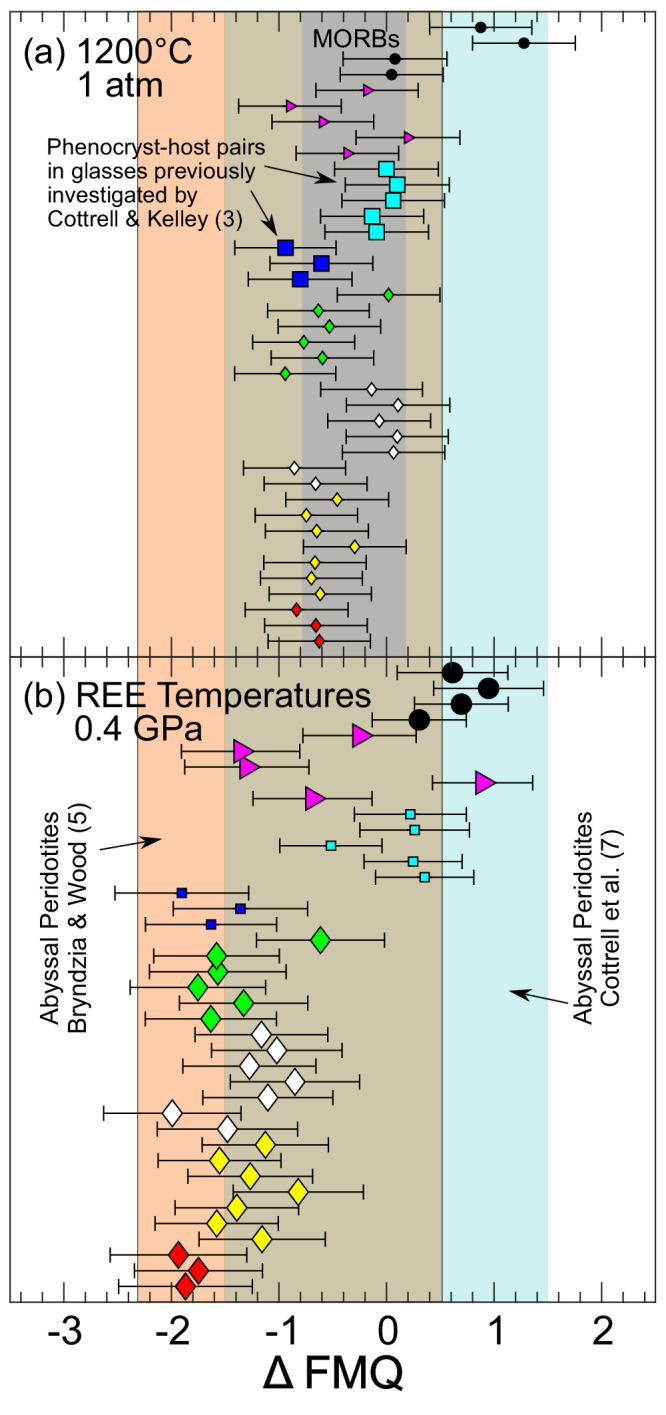
Oxygen fugacities (*f*O_2_s) plotted relative to the fayalite-magnetite-quartz buffer^34^ (Δ FMQ). In (**a**), Δ FMQ values are calculated at 1200°C and atmospheric pressure; in (**b**) they are calculated at 0.4 GPa and their REE temperatures. Phenocryst-host pairs are plotted using larger symbols in (**a**), plagioclase-hosted melt inclusions are plotted using larger symbols in (**b**). Error bars show one sigma uncertainties assessed in Monte Carlo simulations that propagate estimated error for model input parameters through the oxybarometer for each sample ($T$, $P$, major element composition, measured ${D}_{{Eu}}$, *K*, and predicted ${D}_{{{Eu}}^{2+}}$ and ${D}_{{{Eu}}^{3+}}$ uncertainties), and are ±0.57 log units on average (see Methods and Supplementary Figs. S10–S12). Fields in background show global variations in Δ FMQ recovered from mid-ocean ridge basalts (MORBs) (gray, Cottrell et al.^7^, calculated at 1200°C and atmospheric pressure, (**a**)) and abyssal peridotites (blue, 0.6 GPa, Cottrell et al.^7^; orange, 1.0 GPa, Bryndzia and Wood^5^, panels (**a**) and (**b**)). Phenocryst-host pairs are within uncertainty of values previously determined for the same samples by Cottrell and Kelley^3^ by an independent Fe speciation-based method (see Supplementary Fig. S13). Plagioclase-hosted melt inclusions demonstrate a much broader distribution of *f*O_2_s (relative to FMQ) at their plagioclase-glass REE temperatures; the overall distribution of *f*O_2_s is similar to that recovered from abyssal peridotites (also see Fig. 4). To interpret symbol types and colors, see legend to Fig. 1. Comparison of (**a**) and (**b**) highlights the importance o*f* temperature for interpreting *f*O_2_ data relative to a reference buffer.

**Fig. 4:**
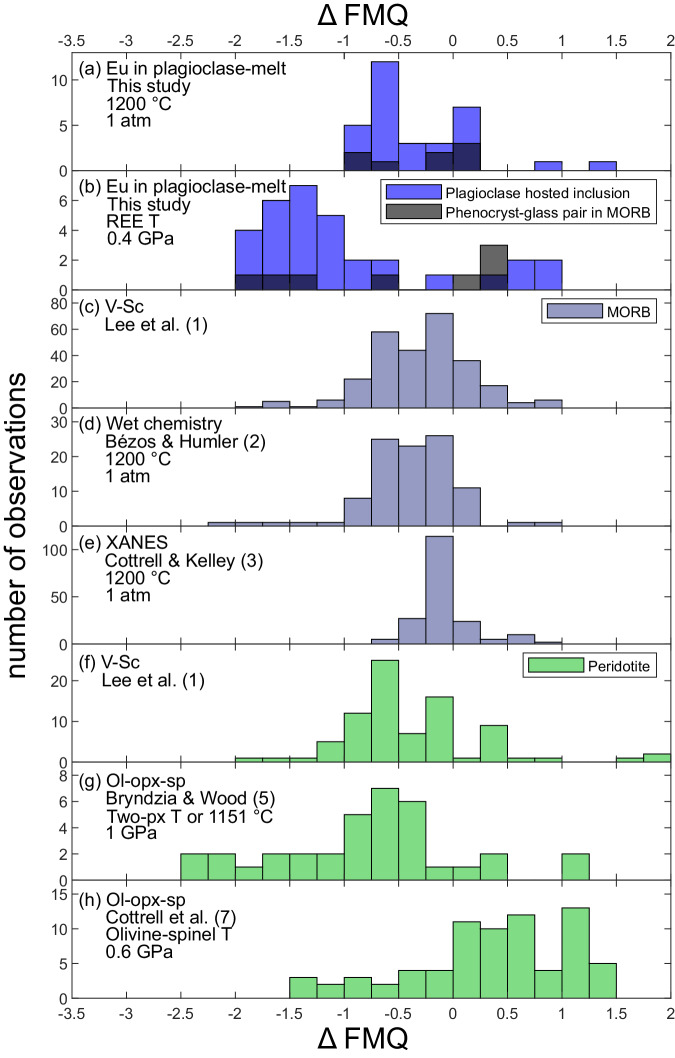
Distributions of oxygen fugacities (*f*O_2_s) relative to the fayalite-magnetite-quartz buffer^34^ (Δ FMQ) calculated at the *P*-*T* conditions indicated in each panel. Data from this study are shown in (**a**) at atmospheric pressure and 1200°C and in (**b**) at 0.4 GPa and their REE temperatures. Plagioclase-hosted melt inclusions are blue and phenocryst-host glass pairs are black. Previous *f*O_2_ determinations for MORBs^1–3^ are shown in (**c**–**e**) (blue gray), and peridotites^1,5,7^ are shown in (**f**)–(**h**) (green). Note the agreement of the breadth of the Eu-in-plagioclase-melt *f*O_2_ distribution and the distribution of peridotites determined using olivine-orthopyroxene-spinel oxybarometry (compare (**b**, **g** and **h**). Oxygen fugacities of phenocryst-host glass pairs determined using Eu distributions are in good agreement with determinations for MORBs using the same $P$-$T$ assumptions (compare black bars in (**a**) to distributions in (**c**–**e**)). When applied to peridotites and MORBs, among all samples, the V-Sc method must assume a single mantle source composition and degree of melting to recover an *f*O_2_, and reference relative to *f*O_2_-dependent partitioning models^76^, which may explain the lesser breadth of the peridotite *f*O_2_s shown in (**f**). Temperatures used in the olivine-orthopyroxene-spinel-based peridotite studies are determined using two-pyroxene solvus thermometers (Two-px Ts) or assumed to be constant (1151 °C) (**g**), or were determined using olivine-spinel thermometry (**h**).

Oxygen fugacities are only reported for plagioclase-glass pairs in apparent equilibrium (as evaluated by the criteria described in the previous paragraph), with the additional stipulation that the samples exhibit smooth and systematic REE patterns in glass and coexisting plagioclase (see Fig. 1 and Supplementary Fig. S8). Oxybarometer inputs include measured Eu distributions (${D}_{{Eu}}$), and ${D}_{{Eu}}^{3+}$ and ${D}_{{Eu}}^{2+}$ calculated using lattice strain-based predictive models^13^ (Eqs. 8–14). The ${D}_{{Eu}}^{3+}$ and ${D}_{{Eu}}^{2+}$ partition coefficient predictions are dependent on plagioclase major element composition, temperature ($T$) and pressure ($P$). The assumed pressure was 0.4 GPa for all samples considered here^25–27^. Two assumed *T*s were used to calculate *f*O_2_s separately, those inverted from the measured trivalent REE distributions using Eqs. 16–19 (the temperature distributions are presented below, and inversions presented in Supplementary Figs. S5–S7), and an assumed $T$ of 1200 °C, which is widely applied in studies that characterized *f*O_2_s of MORBs using other methods^2–4^. Application of Eqs. 7–19 recovers *f*O_2_s summarized in Fig. 3 relative to the FMQ buffer at 0.4 GPa^34^ at 1200 °C in (a) and at the trivalent REE temperatures in (b). The *f*O_2_ distributions are compared to results of previous studies in histograms in Fig. 4.

### Rare earth element in plagioclase-melt temperatures

**Fig. 5:**
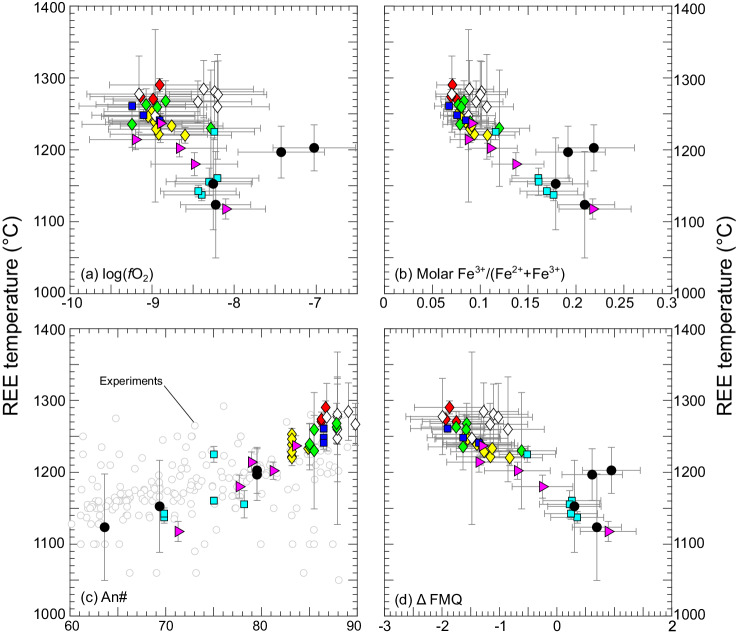
Rare earth element (REE) temperature systematics. Temperatures inverted from plagioclase-glass REE distributions (Eqs. 16–19) are plotted against the log of the oxygen fugacity log(*f*O_2_) recovered from Eqs. 7–19 in (**a**), molar Fe^3+^/(Fe^2+^+ Fe^3+^) in glass calculated using the model of Kress and Carmichael^47^ in (**b**), plagioclase An# (100 × Ca/(Ca + Na + K), in moles) in (**c**), and deviation from the fayalite-magnetite-quartz buffer (Δ FMQ) at 0.4 GPa^34^ in (**d**). Circles in the background of (**c**) are plagioclase-saturated experiments with oceanic basalt and basaltic andesite liquids downloaded from the LEPR database^77^. Error bars for the temperatures are estimated from the uncertainty in the slope in the temperature inversions (Supplementary Figs. S5-S7); error bars for *f*O_2_s are estimated 1σ uncertainties recovered from Monte Carlo simulations (see Methods and Supplementary Figs. S10–S12 for details).

Recovered temperatures range from 1118–1290 °C (Fig. 5), comparable to the range of temperatures among experiments used to calibrate the oxybarometer (1127–1350 °C). In many cases, the full suite of REEs and Y are available for temperature determinations. In some of the literature datasets, only a few element pairs are reported. Among the natural samples, we accepted those that returned magmatic temperatures, regardless of the number of elements available for the temperature inversions. Temperature uncertainties were estimated from uncertainties in the slopes of the temperature inversions (ranging from 7 – 120°C, 26°C on average). The recovered temperatures are consistent with run conditions of plagioclase-saturated oceanic basalt and basaltic andesite experiments at the An#s of the plagioclase investigated in this study (compare gray circles and REE temperatures in Fig. 5c). A MATLAB script is provided as Supplementary Software so that interested readers can calculate REE temperatures using their own data. We note that temperatures determined by application of an independent major element-based plagioclase-liquid thermometer^35^ exhibit a more limited range of ~100°C (Supplementary Fig. S9), but analogous covariation of temperature with An#, comparable maximum temperatures, and qualitatively consistent covariations of temperature with molar Fe^3+^/(Fe^2+^+Fe^3+^) and Δ FMQ.

### Oxygen fugacity systematics

Oxygen fugacities of six plagioclase phenocryst-host glass pairs in four samples from the mid-Atlantic ridge (TR138-9D-4, A11-107-7-20-3, EN025-6D-3, and EN025-2D-4) calculated using the same *T-P* assumption as Cottrell and Kelley (1200 °C, atmospheric pressure), are indistinguishable within error from values determined by iron speciation from the same glasses^3^ (1σ). This supports our assessment of the accuracy of the Eu-in-plagioclase-melt oxybarometer (large squares, Fig. 3a, Supplementary Fig. S13). Two other pairs from sample A11-107-7-20-3 (collected along the mid-Atlantic near the Tristan hotspot) deviate further from the previous determinations, but are within 2σ uncertainty of the previously determined values (Supplementary Data 3; Supplementary Fig. S13). In general, the recovered *f*O_2_s of most samples are within error of results of previous studies that characterized MORBs assuming a constant *T* of 1200 °C (compare Fig. 4a and c–e).

In contrast to the uniformity of the results assuming a constant temperature, calculated *f*O_2_s range from about two log units below to about one log unit above the FMQ buffer when their REE temperatures are applied (Figs. 3b, 4b). They overlap the narrower range of *f*O_2_s determined from Fe speciation in basaltic glasses (gray field, Fig. 3a) and match the distribution of *f*O_2_s recovered from abyssal peridotites using olivine-orthopyroxene-spinel oxybarometry (Fig. 3b, also compare Fig. 4b and g, h). We emphasize the importance of the temperature assumed when a recovered *f*O_2_ is compared to a reference buffer (compare Fig. 5a and d). By necessity (and perhaps reasonably for primitive MORBs), a single temperature is commonly assumed to reference MORBs, while our new data are calculated relative to the FMQ buffer at their trivalent REE temperatures, producing the distribution shown in Figs. 3b and 4b. This distribution is not an artifact of using temperatures from the REE in plagioclase-melt thermometer, as a major element-based plagioclase-melt thermobarometer (Putirka^35^) recovers ranges of temperatures and *f*O_2_s slightly narrower than but comparable to those shown in Figs. 3b, 4b, and 5d (Supplementary Fig. S9).

**Fig. 6:**
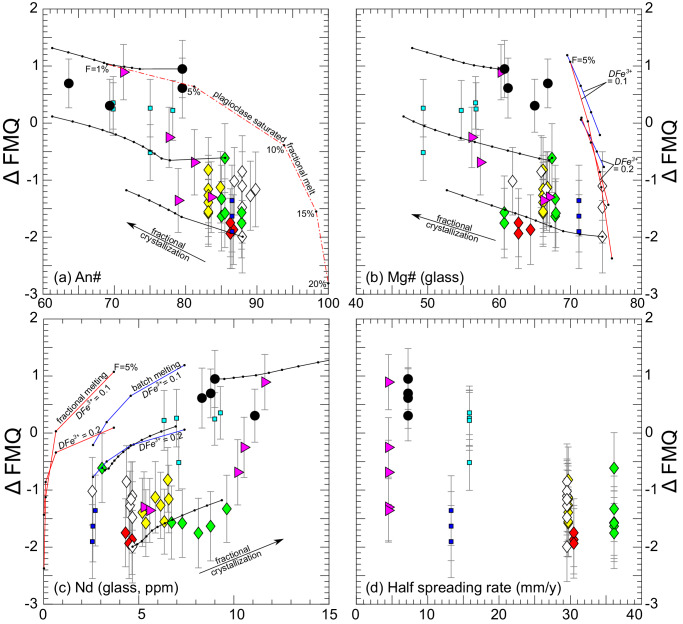
Comparisons of oxygen fugacities (*f*O_2_s) of natural samples to geochemical indices of fractionation, and spreading rates. Simple models for mantle melting and crystal fractionation are also shown. Oxygen fugacities of the natural samples were determined using measured Eu distributions and Eqs. 7–19, and are plotted relative to the fayalite-magnetite-quartz buffer^34^ (Δ FMQ) at 0.4 GPa at their REE temperatures. In (a), Δ FMQ is plotted against plagioclase An# (100 × Ca/(Ca + Na + K), in moles), in (**b**), against glass Mg# (100 × Mg/(Mg + Fe^2+^+ Fe^3+^), in moles), in (**c**), against Nd in glass, in parts per million (ppm), in (**d**), against half spreading rate, in millimeters per year (mm/y). Symbol colors and types are as defined in Fig. 1; larger symbols are melt inclusion data and smaller symbols are phenocryst-host glass pairs. Note the systematic inverse correlation of *f*O_2_ with An# (**a**), Mg# (**b**), and spreading rate (**d**) and positive correlation with Nd (**c**). The trend for other REEs is similar to Nd, with samples exhibiting higher REE concentrations having higher *f*O_2_s. Fractional and batch melting and fractional crystallization models (solid red, blue and black lines respectively) assume *f*O_2_ is modulated by magmatic fractionation of divalent from trivalent iron. Bulk Fe^3+^ partition coefficients assumed in the melting models are indicated. Unless otherwise noted, small dots on the melting and fractional crystallization models are 5% increments (F indicates extent of melting, Eqs. 21–23). Dash-dotted red line in (**a**) shows *f*O_2_s of an instantaneous fractional mantle melt cooled to the point of plagioclase saturation (the melting model assumes bulk a ${D}_{{{Fe}}^{3+}}$ of 0.1). All melting models assume adiabatic decompression of a DMM source^32^ at a potential temperature of 1300°C. Iron species proportions are calculated using the model of Kress and Carmichael^47^ using *f*O_2_s determined from Eu distributions and Eqs. 7–15 at the REE temperatures. Error bars are estimated 1σ uncertainties recovered from Monte Carlo simulations (see Methods and Supplementary Figs. S10–S12 for details).

At their REE temperatures, the experimentally homogenized inclusions mostly produce lower *f*O_2_ values than the other samples, which we speculate is an artifact of sample bias, as they are all compositionally primitive (Figs. 1 and 6). The uncorrected and fractionation-corrected plagioclase-hosted melt inclusions record high *f*O_2_s and Δ FMQs. Correlations of *f*O_2_s (relative to the FMQ buffer) with indices of fractionation (plagioclase An#, glass Mg#, and glass Nd concentration, Fig. 6a–c), with more oxidized samples having more evolved compositions and more reduced samples having more primitive compositions, suggest the distribution of *f*O_2_s is produced by the formation and subsequent evolution of the sample, rather than being an artifact of the sample type. We note there is no evidence for bias in the Eu-in-plagioclase-melt oxybarometer associated with An# or other compositional parameters (Supplementary Fig. S14).

Plagioclase-hosted melt inclusions are entrapped at relatively high lithospheric pressures of ~0.4 GPa during transport to the seafloor^25–27^. Their compositional diversity (Figs. 1, 6a–c) may be interpreted as a record of variable extents of melting not observable in erupted MORBs, which are aggregated increments of near-fractional melting homogenized in melt transport pathways and magma chambers before eruption^8,36,37^, with variably superimposed fractional crystallization. The Mg# of a basaltic liquid increases with partial melting; similarly, the An# of a plagioclase crystal on a basalt liquidus is positively correlated with extent of partial melting (Fig. 1a; 6a and 6b). Incompatible element concentrations in basalts (e.g., Nd) are relatively high at low degrees of melting and progressively decrease as mantle sources are depleted. Canil et al.^38^ and Woodland et al.^39^ observed inverse Fe^3^/Fe^T^ (molar Fe^3+^ / (Fe^3+^ + Fe^2+^)) vs. MgO correlations in peridotite melting residues that could be successfully modeled assuming Fe^3+^ is incompatible during melting, suggesting that Earth’s mantle melts under unbuffered conditions, and demonstrating the potential for the extent of partial melting to affect *f*O_2_s of mantle melts (and residues), by controlling their Fe^3^/Fe^T^ ratios. All else being equal (bulk composition, mineralogy, $T$, and $P$), systems with high Fe^3^/Fe^T^ are more oxidized than systems with low Fe^3^/Fe^T^ ^5,39^. The positive correlations of Nd with *f*O_2_, and inverse correlations of glass Mg# and plagioclase An# with *f*O_2_ are all consistent with a near fractional partial melting control on *f*O_2_ during MORB petrogenesis, produced by fractionation of ferrous from ferric iron (see below for discussion on the effect of fractional crystallization on the *f*O_2_ evolution of a basaltic liquid). Corroborating evidence for control of *f*O_2_ by partial melting comes from application of oxybarometers to minettes and primitive alkali basalts (both produced by low-degree mantle melting), which are highly oxidized relative to *f*O_2_s recorded by higher degree melts of comparable mantle sources, implying an inverse correlation of melt *f*O_2_ with extent of melting^20,40,41^.

If *f*O_2_s of basaltic mantle melts decrease as the extent of partial melting increases, they should increase as fractional crystallization proceeds, as trivalent Fe is incompatible in olivine, pyroxenes, and plagioclase^,42–45^. That trivalent iron is incompatible in fractionating basalt has been inferred from studies of variably fractionated basaltic liquids, which found an inverse correlation of Fe^3+^ with MgO, the latter a proxy for olivine fractionation (e.g., Bézos & Humler^2^, Cottrell & Kelley^3^). Corroborating this interpretation, an analysis of covariations among trace elements in MORBs determined that Fe^3+^ behaves as an incompatible element with a partition coefficient similar to Li during crustal evolution^45^.

How does the oxidation state of Eu track the oxidation state of Fe? Eu is present in basaltic systems in dilute concentrations; it will respond to the partial pressure of oxygen in the system, becoming more oxidized (dominantly Eu^3+^) under high *f*O_2_ conditions or more reduced (dominantly Eu^2+^) under low *f*O_2_ conditions^46^, and its partitioning behavior will respond to a change in *f*O_2_ according to Eq. 6. Thus, Eu distributions should passively record *f*O_2_s imposed by mineral buffers (if they are present), or in an unbuffered basaltic system whose oxidation state is reflected by Fe^3^/Fe^T^, the relative proportions of the reduced and oxidized Fe species.

### Melting and fractionation models

Following the arguments in the previous section, we ran simple partial melting and fractional crystallization models to evaluate the effect of Fe fractionation on *f*O_2_, assuming that Fe^3+^ behaves as an incompatible element and that mantle melting and basalt fractionation are unbuffered. These partial melting and fractional crystallization models are superimposed on *f*O_2_ variations recovered from the natural samples relative to the FMQ buffer in Fig. 6a–c. The simulations employ the model of Kress and Carmichael^47^ (their Eq. 7) to calculate *f*O_2_ as a function of melt composition and Fe^3^/Fe^T^ during melting and fractional crystallization.

Because the *f*O_2_ of a silicate melt depends on $T$ and $P$, as well as major element composition, we used the alphaMELTS software^48,49^ (pMELTS algorithm) to estimate major element variations and $T$ during melting and fractional crystallization, but calculated Mg, Fe, and Nd variations using equations for Rayleigh fractionation and modal batch and instantaneous fractional melting (Eqs. 20–22), as we found that alphaMELTS treated trivalent Fe as effectively compatible or near-compatible during mantle melting, which is not supported by Fe^3^/Fe^T^ systematics of peridotites^38,39^, or measured partition coefficients for mantle silicates^42,44^, or correlations of Fe^3^/Fe^T^ with MgO in fractionated oceanic basalts^2,45^. We used alphaMELTS to calculate mineral modes, plagioclase compositions, and temperature during fractional crystallization of basalts and basaltic melt inclusions, neglecting minor spinel from the fractionation sequences (according to alphaMELTS, spinel accounts for a total of ~1% by mass or less in the fractional crystallization sumulations). Partition coefficient assumptions are reported in Table 1.

**Table 1:** Mineral-melt partition coefficients used in partial melting and fractional crystallization calculations

	${D}_{{{Fe}}^{3+}}$	${D}_{{{Fe}}^{2+}}$	*D*_*Mg*_	*D*_*Nd*_
${D}_{{bulk}}$ (mantle melting)	0.1-0.3	1	3.2	0.03
Olivine (fractional crystallization)	0	1	4	0
Clinopyroxene (fractional crystallization)	0.2	0.6	2	0.1878
Plagioclase (fractional crystallization)	0	0	0	0.065

The evolution of Mg, Fe, and Nd in the liquid during fractional crystallization is calculated using the Rayleigh fractionation equation,

20 $${C}_{l}^{{fract}}={C}_{l0}\times {F}^{({D}_{{bulk}}^{{fract}}-1)},$$

where ${C}_{l}^{{fract}}$ is the compsition of the fractionating liquid, ${C}_{l0}$ is the liquid composition before the onset of fractional crystallization, ${D}_{{bulk}}^{{fract}}$ is the bulk partition coefficient calculated using mineral modes recovered from alphaMELTS simulations. F varies from 1 to 0 as crystallization proceeds.

Fractionation of Mg, Fe, and Nd during mantle fusion is approximated using the equations for modal batch melting^50^

21 $${C}_{l}^{{batch}}=\frac{{{Cs}}_{0}}{{D}_{{bulk}}+F\left(1-{D}_{{bulk}}\right)}$$

and fractional melting^50^

22 $${C}_{l}^{{instantaneous}}=\frac{{{Cs}}_{0}}{{D}_{{bulk}}}{\left(1-F\right)}^{\left(\frac{1}{{D}_{{bulk}}}-1\right)},$$

where ${C}_{l}^{{batch}}$ is the composition of batch melt, ${C}_{l}^{{instantaneous}}$ is instantaneous fractional melt, *F* is the melt proportion, and ${{Cs}}_{0}$ is the initial bulk mantle starting composition (a DMM source^32^ with an assumed initial molar Fe^3^/Fe^T^ of 0.04^38^). Aggregated instantaneous fractional melts are determined using the following expression^50^,

23 $${C}_{l}^{{aggregated}}={{Cs}}_{0}\frac{1-{\left(1-F\right)}^{\left(\frac{1}{{D}_{{bulk}}}\right)}}{F},$$

or approximated as the mass-balanced sum of increments of near-fractional melts when working with outputs of pMELTS simulations.

The bulk ${D}_{{{Fe}}^{2+}}$, ${D}_{{{Fe}}^{3+}}$, ${D}_{{Mg}}$, and ${D}_{{Nd}}$ partition coefficients used in melting models are calculated assuming mineral modes for a DMM source and partition coefficients gathered from the experimental literature^42–44,51–54^. A range of bulk ${D}_{{{Fe}}^{3+}}$ partition coefficients was explored in the melting models, consistent with results of the batch melting experiments of Sorbadere et al.^43^, which determined a bulk peridotite-melt partition coefficient of 0.1–0.3. Partition coefficients employed in fractional crystallization models are gathered from the same literature used to calculate the bulk partition coefficients for the melting models. To model the effect of plagioclase fractionation, we used a partition coefficient from Sun et al.^13^ for Nd and assumed Fe^2+^, Mg, and Fe^3+^ are perfectly incompatible in plagioclase^3^, though there is evidence for more moderate incompatibility of Fe^3+^ from experimental studies^55^. Assuming moderate incompatibility of Fe^3+^ in plagioclase, or a higher (but still < 1) ${D}_{{{Fe}}^{3+}}$ for clinopyroxene makes little difference for the results of the fractional crystallization models as trivalent iron remains incompatible in the fractionating bulk solid. Incompatibility of Fe^3+^ during fractional crystallization of MORBs is supported by covariations of Fe^3+^ and incompatible trace elements^45^, and inverse correlations of Fe^3^/Fe^T^ and MgO in XANES^3^ and wet chemistry-based studies^2^.

Results of the melting and fractional crystallization models are shown in Fig. 6a–c where *f*O_2_s of the modeled melts are plotted relative to the FMQ buffer against indices of fractionation and compared to samples investigated in this study. Fractional crystallization models are shown as black lines with dots indicating 5% solidification increments. Melting models are shown as solid blue and red lines (batch and instantaneous fractional models, respectively), with dots representing 5% melting increments. Cases with bulk ${D}_{{{Fe}}^{3+}}$ of 0.1 and 0.2 are shown for comparison. The dash-dotted line in (a) represents an instantaneous fractional mantle melt cooled to the point of plagioclase saturation.

The fractional melting models approximate Δ FMQ ranges and compositional distributions among natural samples. Trivalent iron is extracted from the mantle source along with incompatible normative albite, producing the inverse correlation of plagioclase An# with *f*O_2_ shown in Fig. 6a. Similarly, Fe^3+^ is extracted from the mantle source as melting proceeds while compatible Mg is retained such that fractional melts become progressively enriched in Mg relative to Fe, producing the inverse correlation of Δ FMQ and Mg# shown in (b). A correlation in the opposite sense is shown in (c), where melting progressively depletes the mantle source in Nd, along with Fe^3+^, producing lower *f*O_2_s at higher extents of melting. The breadth of the compositional distributions is explained by fractional crystallization superimposed upon the melting process (several examples are shown as solid black lines).

Because these partial melting and fractional crystallization models assume constant, *P*- and *T*-insensitive Fe^3+^ partition coefficients within the bounds of recent experimental determinations^42–44,54^, the model results are schematic. Nonetheless, the models broadly reproduce the *f*O_2_ distributions of the natural samples and their correlations with fractionation indices. We recognize that in reality, Fe speciation and partitioning vary according to *f*O_2_, $T$, $P$, crystal chemical, and phase equilibria determinants all affected by the process of decompression melting^56,57^, but the consistency of our simplified models with the observed *f*O_2_ distributions suggests that they capture Fe’s first-order behavior, and its effect on magmatic *f*O_2_, during melting and fractional crystallization.

Gaetani^56^ developed a model for the evolution of *f*O_2_ during partial melting that considered point defect concentrations in olivine, and olivine-spinel-pyroxene equilibria, and concluded that the *f*O_2_ of a basaltic melt depends on the potential temperature of melting, with higher potential temperatures producing more reduced melts. The predictions of Gaetani’s model are qualitatively consistent with our results, if higher potential temperatures correlate with greater extents of melting, but the three order of magnitude range in Δ FMQ recovered from plagioclase-hosted melt inclusions is much larger than the one order of magnitude difference predicted for reasonable potential temperature maxima and minima according to the model of Gaetani. From our results and models, we infer that loss of Fe^3+^ from a mantle source during near-fractional melting must reduce the source even as $P$ and $T$ vary and the source reequilibrates, because trivalent iron is incompatible in MORB-source peridotite^43^, and mass must be conserved.

**Fig. 7:**
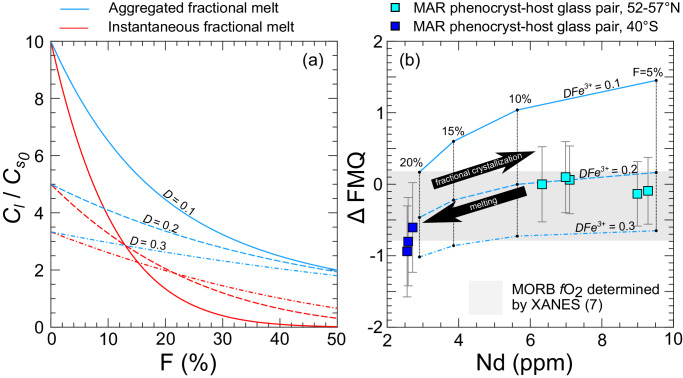
Schematic demonstration of the potential for partial melting to control the oxygen fugacity (*f*O_2_s) of mid-ocean ridge basalt (MORB). Shown in (**a**) are relationships between instantaneous fractional melts and aggregated fractional melts for partition coefficients (*D*s) relevant to bulk mantle Fe^3+^ partitioning^43^. Shown in (**b**) are *f*O_2_s of modeled mantle melts, assuming basalts are the product of aggregated near-fractional melting, compared to *f*O_2_s recovered from natural samples (squares). In (**a**), abundances of elements in liquids produced by melting (${C}_{l}$) are normalized by the initial concentration in the solid (${{Cs}}_{0}$), and plotted as a function of extent of melting (F) (Eqs. 22 and 23). In (**b**), *f*O_2_ is shown relative to the fayalite-magnetite-quartz buffer^34^ (Δ FMQ), calculated at atmospheric pressure and 1200 °C, plotted against Nd concentration in glass (or melt) in parts per million (ppm). Squares are *f*O_2_s recovered from Eu distributions in phenocryst-host pairs from mid-Atlantic ridge (MAR) basalts. Aggregated fractional melts of a DMM^32^ source (blue lines) assume bulk trivalent Fe partition coefficients (${D}_{{{Fe}}^{3+}}$) of 0.1–0.3 and an initial mantle Fe^3^/FeT of 0.04. Gray field in background shows the range of *f*O_2_s in MORB glasses determined by an iron speciation-based method^7^ (X-ray absorption near edge spectroscopy, XANES). The *f*O_2_s and Nd abundances of the phenocryst-host glass pairs can be successfully modeled as aggregated near fractional melts of a DMM source with a bulk mantle ${D}_{{{Fe}}^{3+}}$ of ~0.2. Fractional crystallization of the aggregated melts would produce a moderate increase of Δ FMQ with Nd sub-parallel to the melting trends (shown schematically by the black arrow, see Fig. 6c for sample-specific examples). Error bars in (**b**) are estimated 1σ uncertainties recovered from Monte Carlo simulations (see Methods and Supplementary Fig. S11 for details).

Consistency in *f*O_2_ between the basaltic melt inclusions and abyssal peridotites (Figs. 3 and 4), and our ability to successfully model covariation of *f*O_2_ with mantle melting and fractional crystallization (Fig. 6), suggest unbuffered magmatic processes control *f*O_2_s of near-fractional basaltic mantle melts by modulating Fe^3^/Fe^T^. Oxygen fugacities recorded by plagioclase-hosted melt inclusions and MORBs suggest the MORB source has a bulk Fe^3^/Fe^T^ of 0.04, if the bulk trivalent iron partition coefficient is ~0.2 (Figs. 6 and 7).

### Reconciling the *f*O_2_s of MORBs and plagioclase-hosted melt inclusions

Calculated at 1200 °C and atmospheric pressure, the *f*O_2_s of plagioclase-hosted melt inclusions are similar to the distributions of *f*O_2_s recovered from MORBs by wet chemistry, and V-Sc-based methods, and broader than, but mostly still within uncertainty of the XANES-based determinations (Figs. 3 and 4). In contrast, at the melt inclusion entrapment conditions suggested by the REE temperatures, the plagioclase-hosted melt inclusions exhibit a three order of magnitude distribution of *f*O_2_s consistent with peridotites. The broader distribution of melt inclusion *f*O_2_s can be reconciled with the relative *f*O_2_ uniformity of MORBs by recognizing that MORBs are aggregates of near-fractional mantle melting^8,36,37^, whereas plagioclase-hosted melt inclusions are more representative of increments of near-fractional melting^30^.

As Fe^3+^ is incompatible during mantle melting^43^, it will be depleted in the source as melting progresses, such that near-fractional melt contains progressively less Fe^3+^ as the extent of melting increases (shown schematically in Fig. 7a, red lines). The Fe^3+^ accumulates in the aggregated basaltic liquid (blue lines, Fig. 7a), and is at its maximum with the first increment of melting. As melting proceeds, the initially Fe^3+^ rich aggregated liquid is diluted by addition of Fe^3+^ depleted melt. For extents of melting relevant to MORB petrogenesis (*F* = 5–20%), the effect of extent of melting on the Fe^3+^ abundance in the aggregated liquid is minor if the bulk partition coefficient of Fe^3+^ is between 0.2 and 0.3^43^ (dashed and dash dotted blue lines, Fig. 7a).

Assuming bulk Fe^3+^ partition coefficients of 0.1−0.3 and a DMM source with an Fe^3^/Fe^T^ of 0.04, we modeled aggregation of fractional mantle melts using Eq. 23 and the near-fractional melting outputs of pMELTS simulations for isentropic melting at a mantle potential temperature of 1300°C. We then applied the model of Kress and Carmichael^47^ to calculate *f*O_2_s of the aggregated fractional liquids (see Melting and Fractionation Models for details), referenced to the FMQ buffer at atmospheric pressure and 1200°C^34^. The melting models are in good agreement with *f*O_2_s recovered from phenocryst-host glass pairs using the Eu-based oxybarometric method (Eqs. 7–19) assuming a bulk Fe^3+^ partition coefficient of 0.2 (compare dark and light blue squares to dashed line in Fig. 7b). Fractional crystallization would produce a moderate increase of *f*O_2_ with Nd, as shown schematically by the black arrow^2,45^. The success of these models in reproducing our data, and the *f*O_2_s of MORBs recovered using several independent methods (Figs. 3, 4b, 5–6) suggests MORB *f*O_2_s reflect homogenization (aggregation) of heterogeneous, near fractional melts in subaxial magma chambers that originate from a DMM with an average *f*O_2_ approximating the FMQ buffer (Fig. 7), with whatever additional effects open system processes (e.g., degassing, assimilation, crystal fractionation, magma chamber recharge events), and charge transfer upon quenching superimpose upon MORBs and MORB glasses.

### Relationships between oxygen fugacities of peridotites and plagioclase-hosted melt inclusions and implications for Earth’s convecting mantle

The overlapping *f*O_2_s of plagioclase-hosted melt inclusions and abyssal peridotites (Figs. 3b, 4b, 4g, 4h) may suggest a shared evolutionary history, but evaluation of the processes that affect distributions of peridotite *f*O_2_s is required. Abyssal peridotites are often extensively affected by low-temperature serpentinization, which produces an oxidized residue. However, a systematic study of *f*O_2_s of variably altered abyssal peridotites found negligible influence of serpentinization on *f*O_2_s recovered using olivine-orthopyroxene-spinel oxybarometry^58^, as the phases in the peridotites analyzed for application in the oxybarometric method were chemically undisturbed.

The $P$-$T$ changes associated with the tectonic exhumation and unroofing of abyssal peridotites affect *f*O_2_s. Temperatures recorded by abyssal peridotites using olivine-spinel thermometers are consistent with subsolidus conditions (~800–1100 °C)^59^ rather than magmatic conditions. Several studies^9,56,57^ projected peridotites from lower to higher $P$-$T$ conditions, finding decreases in *f*O_2_ relative to the FMQ buffer with increasing $P$-$T$. These projections are non-trivial, as a proper treatment requires $T$ and $P$ sensitive reconstruction of mineral compositions and mineral modes, as well as accounting for the $T$ and $P$ sensitivity of a reference buffer. Stolper et al.^57^ (at constant temperature) highlighted the effect of pressure-dependent aluminous phase stability on trivalent Fe activity, and thereby, *f*O_2_, and found a decrease in *f*O_2_ with pressure increasing from the plagioclase stability field to the spinel stability field. For a suite of abyssal peridotites from the SWIR^9^_,_ Birner and coauthors estimated that under subsolidus conditions, *f*O_2_s of the peridotites are about 0.6 log units higher than they were at magmatic $P$-$T$ conditions. These models suggest the distribution of subsolidus peridotite *f*O_2_s might shift to lower values when corrected to $P$-$T$ conditions relevant to partial melting, but the breadth of the distribution of *f*O_2_s recorded by peridotites is not an artifact of their thermal or pressure evolution.

Taking this into account, we infer that the plagioclase-hosted melt inclusions record *f*O_2_s similar to the peridotites because the inclusions were produced by peridotite melting, and the near-fractional melts and melting residues were in equilibrium at the time of melting. If near-fractional melting decreases mantle *f*O_2_, why are some peridotites more oxidized than MORBs and most plagioclase-hosted melt inclusions? Metasomatic or melt impregnation events by low degree, high Fe^3^/Fe^T^ melts may elevate peridotitic *f*O_2_s initially near or below the FMQ buffer^60,61^. Assuming spreading centers with slower spreading rates produce proportionally more plagioclase-hosted melt inclusions comprising lower degree melts^62^, production of high *f*O_2_ melts by low degree mantle melting may be demonstrated by a scattered inverse correlation of Δ FMQ with half spreading rate (Fig. 6d), though application of the Eu-in-plagioclase-melt oxybarometer to a more global sample suite that is experimentally treated and analyzed in the same way as the inclusion-bearing plagioclase phenocrysts is needed to evaluate this interpretation. Perturbation of peridotitic *f*O_2_s by interactions with oxidized low degree mantle melts may have a minor effect on *f*O_2_ except in cases of exceptional melt:rock ratios^63^.

If partial melting and melt-rock interaction events do not affect the *f*O_2_s of abyssal peridotites, their broad *f*O_2_ distribution must reflect a MORB source inherently heterogeneous in *f*O_2_ over length scales short enough to be averaged by transport pathways that focus and deliver mantle melts to spreading centers^9,64–66^. However, in that case, whatever process produces variable *f*O_2_s must also produce systematic variations in major and trace element indices of fractionation (Fig. 6a–c).

The apparent efficiency of mantle reduction during partial melting requires that melting is effectively unbuffered over the extents of melting that occur beneath seafloor spreading centers. Applications of paleoredox proxies to Paleoproterozoic and even Archean and Hadean to modern samples suggest the oxidation state of Earth’s convecting mantle has been similar to the modern value throughout much of geologic time^67–70^. The *f*O_2_ of the modern mantle is moderated by both melting and subduction, which delivers oxidized materials back into the asthenosphere. Assuming Fe was analogously fractionated by mantle melting in the past, the lack of secular variation in mantle *f*O_2_ since at least the Paleoproterozoic implies that plate tectonic cycling (perhaps with contributions from other mechanisms, e.g., lithospheric delamination) provided an influx of oxidized material into a mantle otherwise occupied by reduced domains produced by partial melting, and that the subducted materials were not effectively reduced as they passed beneath magmatic arcs (e.g., by melting or degassing), which would upset the *f*O_2_ balance. Relatively oxidized, subducted MORB source materials and correspondingly depleted mantle residues were effectively mixed (though perhaps not chemically homogenized) over spatial scales smaller than MORB source regions prior to subsequent melting events.

## Methods

### Equilibrium constant determination

The equilibrium constant ($K$) was determined by fitting ${D}_{{Eu}}$ as a function of *f*O_2_ using Eq. 6, where *f*O_2_ is experimentally imposed and ${D}_{{{Eu}}^{2+}}$ and ${D}_{{{Eu}}^{3+}}$ are predicted using the composition, *T* and *P* dependent lattice strain-based partitioning models of Sun et al. ^13^ (Eqs. 8–14). Fitting $K$ using ${D}_{{Eu}}$ as the dependent variable (Eq. 6) produced a better fit than using *f*O_2_ as the dependent variable (Eq. 7), as *f*O_2_s determined using Eq. 7 are sensitive to small variations in ${D}_{{Eu}}$ under very reducing or very oxidizing conditions (as in experiments conducted in air, where ${D}_{{Eu}}$ approaches ${D}_{{{Eu}}^{3+}}$), such that outlying data skew the fit (e.g., Supplementary Fig. S3). $K$ was determined by a simultaneous nonlinear least squares regression of the experimental observations, which include 42 ${D}_{{Eu}}$ measurements from experiments testing terrestrial basaltic systems. The quality of the fit is shown in Supplementary Fig. S1a. Uncertainty in the equilibrium constant was calculated from the distribution of residuals about the mean (Supplementary Fig. S1b).

Experimental *f*O_2_s predicted using Eq. 7 are shown in Supplementary Fig. S2, with calibrating observations plotted as colored symbols and an extrapolation dataset shown as gray dots (dacites, planetary systems, and simple synthetic systems). The model recovers experimentally imposed *f*O_2_s from measured ${D}_{{Eu}}$ values for most experiments, except at very high *f*O_2_s (experiments conducted in air) where the oxybarometer becomes *f*O_2_ insensitive (Supplementary Fig. S3).

### Experimental homogenization

Homogenization experiments from Lewis et al.^28^ were conducted at 1 bar using the vertical quench furnace at South Dakota School of Mines and Technology. Experiments followed the procedure used by Nielsen et al.^29^ and were performed in air at 1230 °C without measurement of *f*O_2_. The run temperature was selected after observation of inclusion-hosted crystal melting in experiments heated sequentially^71^. Batches of crystals were heated to just below their estimated entrapment temperature and examined for the presence of inclusion-hosted crystals and the degree to which the melt inclusion lay at or near the olivine-plagioclase cotectic. If daughter crystals or sidewall crystals were present in the melt inclusion, another batch of crystals was heated to a temperature 10°C above the initial batch. This process was continued until the melt inclusion compositions departed from the olivine-plagioclase cotectic onto a plagioclase control line. These temperatures were then confirmed in additional heating stage experiments^27,72^. Depending on the size of the plagioclase host, up to six inclusion-bearing crystals were placed in Pt-foil boats wrapped in fine Pt-quench wire (0.003 inches) and suspended from a thicker Pt-suspension wire (0.024 inches). The run assembly was then lowered into the furnace to the hot spot at 1230°C for the duration of each experiment, which ranged from 30 min to 192 h (Supplementary Data 3). At the end of the designated time, the sample was quenched by running an electric current through the suspension wire, dropping the sample into water. For plagioclase-hosted inclusions, this rapid quench is necessary because crystallization within the inclusion can happen even during the removal of the assembly from the furnace if the sample is cooled in air. Previous work showed that 3 to 5 s between the removal of the run assembly from the furnace and quench produces weight percent-level changes in the Al and Ca concentrations of the melt inclusion^71^.

Run products were optically investigated; melt inclusions that retained daughter crystals were eliminated from further study. Following the homogenization experiments, samples were prepared in 1” round epoxy mounts, then polished to expose the melt inclusions at the surface. The translucent epoxy mounts enable identification of inclusions within unexposed, deeper parts of the crystal using transmitted light microscopy. Electron microprobe analysis^28,29^ provided another means to evaluate the fidelity of the inclusions to their entrapment conditions; only those with major element compositions along the olivine-plagioclase cotectic (rather than plotting in the plagioclase only field) were investigated here.

### Major element analysis

Major elements in melt inclusions and their plagioclase hosts were analyzed at Oregon State University^28,29^. Major elements in mid-Atlantic ridge phenocryst-host pair samples (A11-107-7-20; TR138-9D; EN025-6D; EN025-2D) were measured using a Cameca SX 100 electron microprobe at the University of Tennessee at an accelerating voltage of 15 kV. Plagioclase was analyzed at a current of 10 nA using a 5 μm spot, with count times of 20 s on peak for Si, Ti, Al, Na, and K, 30 s on peak for Ca, and 60 s on peak for Fe. Glass was analyzed at 20 nA with a 15 μm spot, using count times of 20 s for Si, Al, Mg, Ca, S, Na, and K, and 30 s for P, Ti, Fe, Mn, and Cr. Background count times were 15 s. Natural and synthetic reference materials were employed, and data were processed using a ZAF correction applied using the Cameca PAP procedure. Results are reported in Supplementary Data 1.

### Trace element analysis

Trace element concentrations in plagioclase phenocryst-glass pairs (A11-107-7-20; TR138-9D; EN025-6D; EN025-2D) and experimentally homogenized melt inclusions and their plagioclase host crystals (MI18-6; MI18-1; MI19B-4; MI19B-8) were quantified in 1” round epoxy mounts using an Elemental Scientific NWR193 excimer laser system coupled to an Agilent 7500ce inductively coupled mass spectrometer at the University of Texas at Austin. The laser system is equipped with a large format two-volume laser cell with fast washout (<1 s), that accommodated all samples and standards in a single loading. Laser ablation parameters for spot analysis were optimized for sensitivity and signal stability using test ablations on representative unknowns. A range of laser spot sizes were required to maximize coverage within target grains. Prior to analysis, sample target grains and standards were pre-ablated (3.85 J/cm^2^ fluence, 2 Hz, 1 s dwell) to remove potential surface contamination. Sample spot analyses were bracketed hourly by reference materials (USGS BCR2G, NIST 610, NIST 612), measured in triplicate for 45 s. All analyses of standards and target grains were conducted at the same spot sizes, involving runs at 30, 40, 50, 60, 75, 100, and 125 µm diameter spots. Oxide production, as monitored during tuning on NIST 612, averaged 0.31%. Laser energy densities over all analytical runs over 2 days averaged 6.05 ± 0.44 J/cm^2^.

The quadrupole time-resolved method measured 16 masses using integration times of 10 ms (^24^Mg^, 29^Si^, 43^Ca^, 48^Ti), 25 ms (^89^Y, ^139^La, ^140^Ce, ^141^Pr) and 40 ms (^146^Nd, ^147^Sm, ^153^Eu, ^157^Gd, ^159^Tb, ^163^Dy, ^166^Er, ^172^Yb). The sampling period of 0.4912 s corresponds to 93.6% quadrupole measurement time and 152 duty cycles per 75 s dwell time. Time-resolved intensities were converted to concentration (ppm) equivalents using Iolite software^73^, with ^29^Si as the internal standard and a Si index values (wt% Si) assigned from electron microprobe measurements. Dwell intervals, edited to exclude transitions and secondary phases encountered during ablation, ranged from 6 to 75 s with an overall average of 60 ± 21 s (*n* = 192); shorter intervals generally correspond to smaller spot sizes. Baselines were determined from 45 s gas blank intervals measured while the laser was off and all masses were scanned by the quadrupole. The data were processed using NIST 610 as the primary reference standard and accuracy and precision were proxied from replicates of NIST 610 and BCR2G analyzed as unknowns; results are reported in Supplementary Data 5. Secondary standard recoveries relative to GeoREM preferred values^74,75^ improve with larger spot sizes, regardless of the primary calibration standard used. On average the secondary standard recoveries deviate 1.9% and 5.9% from the preferred values (NIST 610 and BCR2G, respectively), see Supplementary Data 5.

### Eu maps

Maps were made using data collected at 5.6 J/cm^2^ fluence, 30 Hz, 10 × 10 µm square aperture, 75 µm/s scan rate, with 2 s between lines to capture baseline values. He and Ar flows were 800–850 mL/min. The method scanned 4 masses (29, 137, 151, 153) with 30 ms integration times. The duty cycle was 0.1322 s, corresponding to 91% measurement time, and a measurement every 9.915 µm (slightly less than the aperture). Standards (NIST 612, 610) were analyzed in duplicate at the beginning and end of maps, or also in the middle for large maps. Iolite4 was used to convert intensities to ppm concentrations, using ^29^Si as the index value and 23 wt% Si as the internal standard value. Ba and Eu recoveries on NIST 610 (against 612 as calibration standard) were within 2% of reference values.

### Method for estimating uncertainty in recovered *f*O_2_s

Uncertainties were estimated independently for each sample from standard deviations of *f*O_2_ distributions recovered after propagation of synthetically perturbed datasets through Eq. 7. Oxybarometer inputs (${D}_{{Eu}}$, *K*, *P*,*T*, ${D}_{{{Eu}}^{2+}}$, and ${D}_{{{Eu}}^{3+}}$, Eqs. 7–15) were perturbed simultaneously by randomized normally distributed errors scaled by the error weights reported in Table 2. One thousand simulations for each sample were propagated through Eq. 7, and the standard deviations of the resulting distributions of *f*O_2_s were calculated (Supplementary Figs. S10–S12). These standard deviations are presented in the figures in the main text and Supplementary Data 3 as the *f*O_2_ uncertainties. The mean uncertainty is ±0.57 log units, with maxima and minima of 0.68 and 0.43 log units. The variability among samples reflects differences in the way the errors propagate for different plagioclase compositions and temperatures.

**Table 2:** Weights assigned to each oxybarometer input during error propagation analysis

Major element composition	5% of the measured concentration for every oxide
${D}_{{Eu}}$	10% of the measured Eu partition coefficient
Equilibrium constant (*K*)	1.115 × 10^-4^ (1σ uncertainty determined from fit residuals)
Pressure	0.2 GPa
Temperature	Uncertainty from inversion (5–120 °C, mean 26 °C)
${D}_{{{Eu}}^{3+}}$ and ${D}_{{{Eu}}^{2+}}$ predictions	Coefficient uncertainties reported in Sun et al.^13^(1σ)

### Supplementary information

Supplementary Information

Description of Additional Supplementary Files

Supplementary Data 1

Supplementary Data 2

Supplementary Data 3

Supplementary Data 4

Supplementary Data 5

Supplementary Software

## Data Availability

The analytical and calculated oxygen fugacity data generated in this study have been deposited in the Zenodo database at 10.5281/zenodo.10934344. The analytical and calculated oxygen fugacity data are also provided as Supplementary Data to the article.

## Code availability

Supporting code is provided in the Electronic Supplement and in the Zenodo repository at 10.5281/zenodo.10934344.
