# Supplementary material for: Europium in plagioclase-hosted melt inclusions reveals mantle melting modulates oxygen fugacity
Source: Nat Commun. 2024 Apr 8;15:3033. doi: 10.1038/s41467-024-47224-5 (PMC11001916; doi:10.1038/s41467-024-47224-5)
Supplement: Supplementary file 1 — Supplementary Information [file 41467_2024_47224_MOESM1_ESM.pdf]

Electronic Supplement to

Europium in plagioclase-hosted melt inclusions reveals mantle melting modulates oxygen fugacity

Nicholas Dygert[1*], Gokce K. Ustunisik[2,3], Roger L. Nielsen[2]

[1]Department of Earth, Environmental and Planetary Sciences, University of Tennessee, Knoxville, TN, 37996

[2]Department of Geology and Geological Engineering, South Dakota School of Mines and Technology, Rapid City, SD, 57701

[3]Department of Earth and Planetary Sciences, American Museum of Natural History, New York, NY, 10024

*Corresponding author. E-mail: ndygert1@utk.edu

Contents

**Figure S1**	Eu partition coefficients predicted using Eqs. 6 and 8-14 and the equilibrium constant (K) determined from experimental observations (Eq. 15), and residuals to the fit
**Figure S2**	Demonstration of the Eu-in-plagioclase-melt oxybarometer's ability to reproduce experimentally imposed fO_2s
**Figure S3**	Calculated Eu partition coefficients plotted as a function of fO_2 for three experiments
**Figure S4**	Temperatures inverted from measured plagioclase-melt REE distributions in experiments compared to experimental temperatures
**Figures S5-S7**	Temperature inversion diagrams for measured plagioclase-melt REE distributions in natural samples
**Figure S8**	Partition coefficients calculated using the measured REE concentrations
**Figure S9**	Covariations of $Fe^{3+}/(Fe^{2+}+Fe^{3+})$, An#, and FMQ with temperatures determined using Putirka's plagioclase-liquid equilibria thermobarometer[1]
**Figures S10-S12**	Histograms showing results of Monte Carlo simulations used to calculate fO_2 uncertainty
**Figure S13**	Demonstration of the Eu-in-plagioclase-melt oxybarometer's ability to recover fO_2s in natural samples previously determined using XANES-based Fe speciation data
**Figure S14**	Lack of compositional correlations with experimental fO_2 determined by Eu-in plagioclase oxybarometry
**Figure S15**	Lack of correlation between fO_2 and experimental homogenization time
**References**	

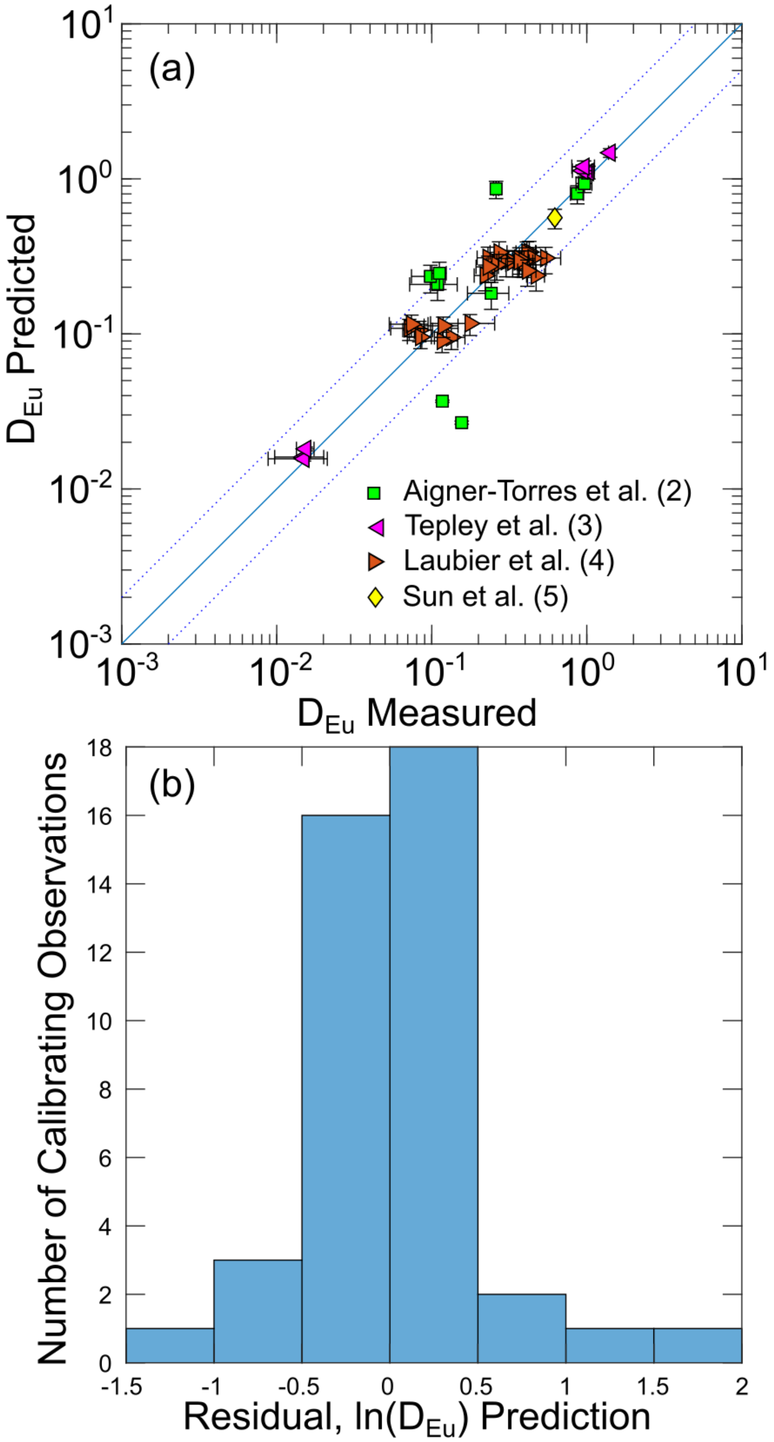

Fig. S1. Eu partition coefficients (D_{Eu}) predicted using the equilibrium constant (K) determined from application of Eqs. 6 and 8-14 to experimental observations from terrestrial basaltic systems[2-5] (Eq. 15, see Methods). Predicted Eu partition coefficients are mostly within uncertainty of the calibrating observations (a); vertical error bars are estimated from the uncertainty in the equilibrium constant, horizontal error bars are reported in the literature. Residuals from the fit exhibit a normal distribution (b) from which the uncertainty in the equilibrium constant is determined.

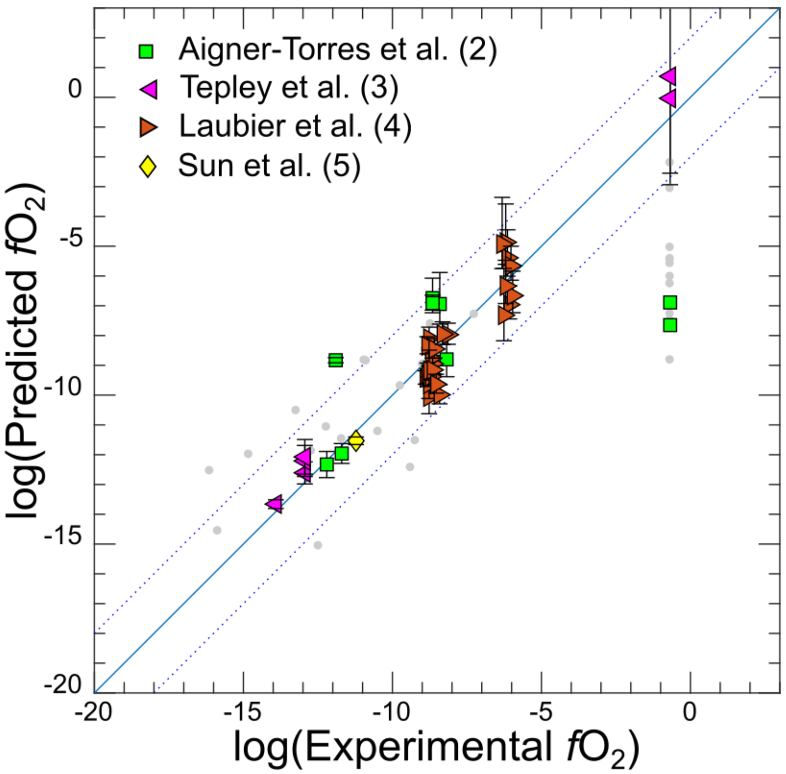

Fig. S2. Demonstration of the Eu-in-plagioclase-melt oxybarometer's ability to reproduce experimentally imposed oxygen fugacities (fO_2s). Colored symbols represent experiments conducted on terrestrial basaltic systems that were used to determine the equilibrium constant in the present study[2-5]. Gray dots in background show model extrapolation to experimental data from simple, evolved, and planetary-relevant systems excluded from the equilibrium constant determination[6-12]. Error bars are calculated using the uncertainty in the equilibrium constant *K* (Supplementary Figure S1b, see Methods for details on the more comprehensive approach used to calculate uncertainty for natural samples). The oxybarometer accurately recovers most experimentally imposed fO_2s in the calibration and extrapolation datasets over geologically relevant fO_2s. See Supplementary Fig. S3 for an explanation of the deviation of predicted from experimental fO_2s for experiments conducted in air.

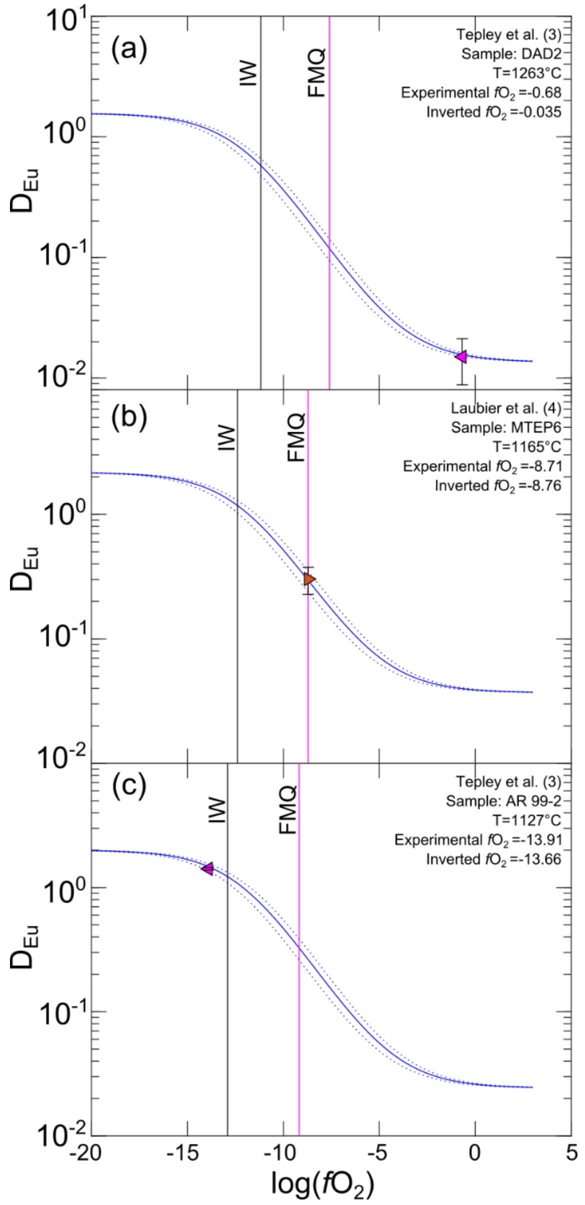

Fig. S3. Calculated Eu partition coefficients (D_{Eu}) plotted as a function of oxygen fugacity (fO_2) using Eqs. 6 and 8-14 (solid blue curves) compared to measured Eu partition coefficients at the experimentally imposed fO_2s (triangles). Panels (a), (b), and (c) each show an individual experimental result and the corresponding D_{Eu} prediction. Experimental conditions, the experiment name, and the data sources are provided in the panels. Dotted curves show D_{Eu} uncertainty propagated from the uncertainty in the equilibrium constant (K). Error bars on the symbols are reported in the experimental literature. Black and magenta lines show the iron-wüstite[13] (IW) and fayalite-magnetite-quartz[14] (FMQ) buffers at the experimental temperature and atmospheric pressure. Note the steep slope of the D_{Eu} curve within ~±5 log units of the FMQ buffer, which helps mitigate error propagation associated with uncertainty in the measured Eu partition coefficient at those fO_2s. Flattening of the D_{Eu} curve at high and low fO_2s (where D_{Eu} approaches divalent and trivalent Eu partition coefficients, respectively) makes the Eu-in-plagioclase-melt oxybarometer susceptible to an unreliable result at those conditions.

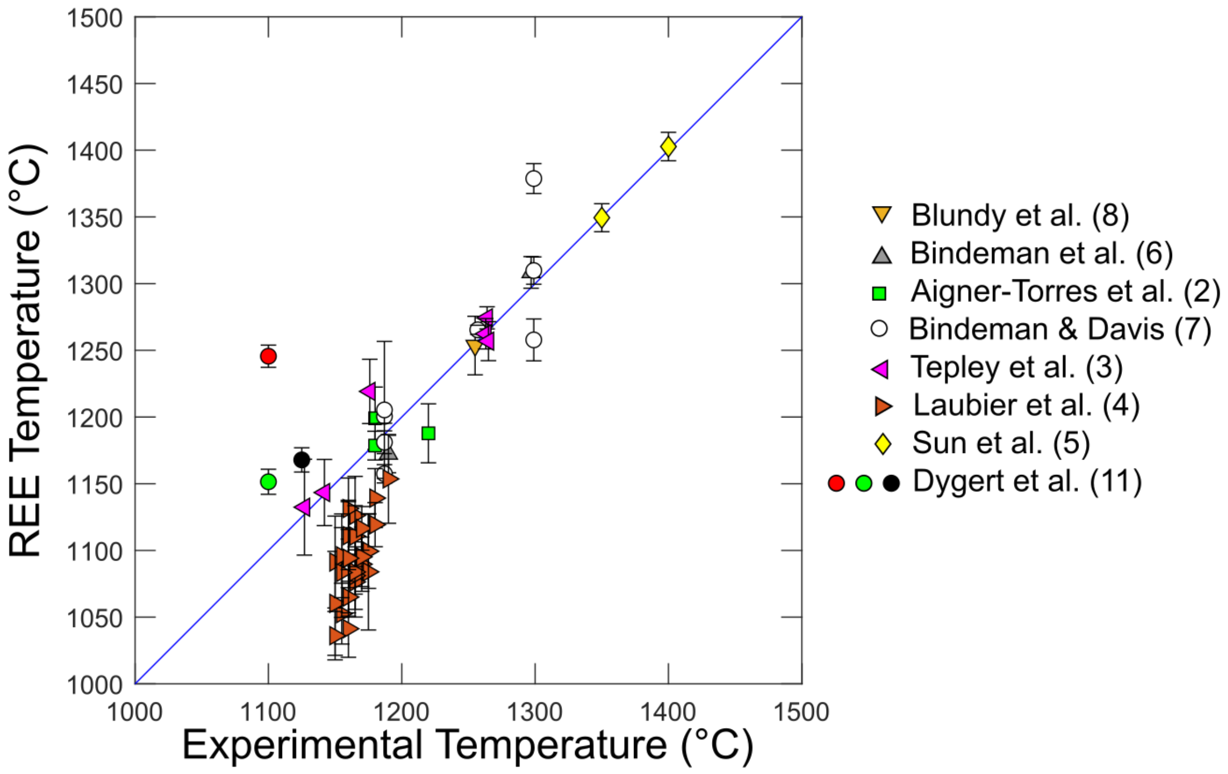

Fig S4. Temperatures inverted from trace element partitioning experiments that exhibit linear REE partition coefficient distributions in temperature inversion space (e.g., Supplementary Fig. S5) (y axis) plotted against experimental temperature (x axis). Uncertainty shown by the error bars is determined from the slope of the line in the temperature inversions (see Supplementary Figs. S5-S7). With the exception of the data of Laubier et al.[4] (which reported 2-4 REE partition coefficients for each experiment, excluding Eu) and a few outliers from other studies[7,11], the thermometer systematically recovers the experimental temperatures from the measured partitioning data.

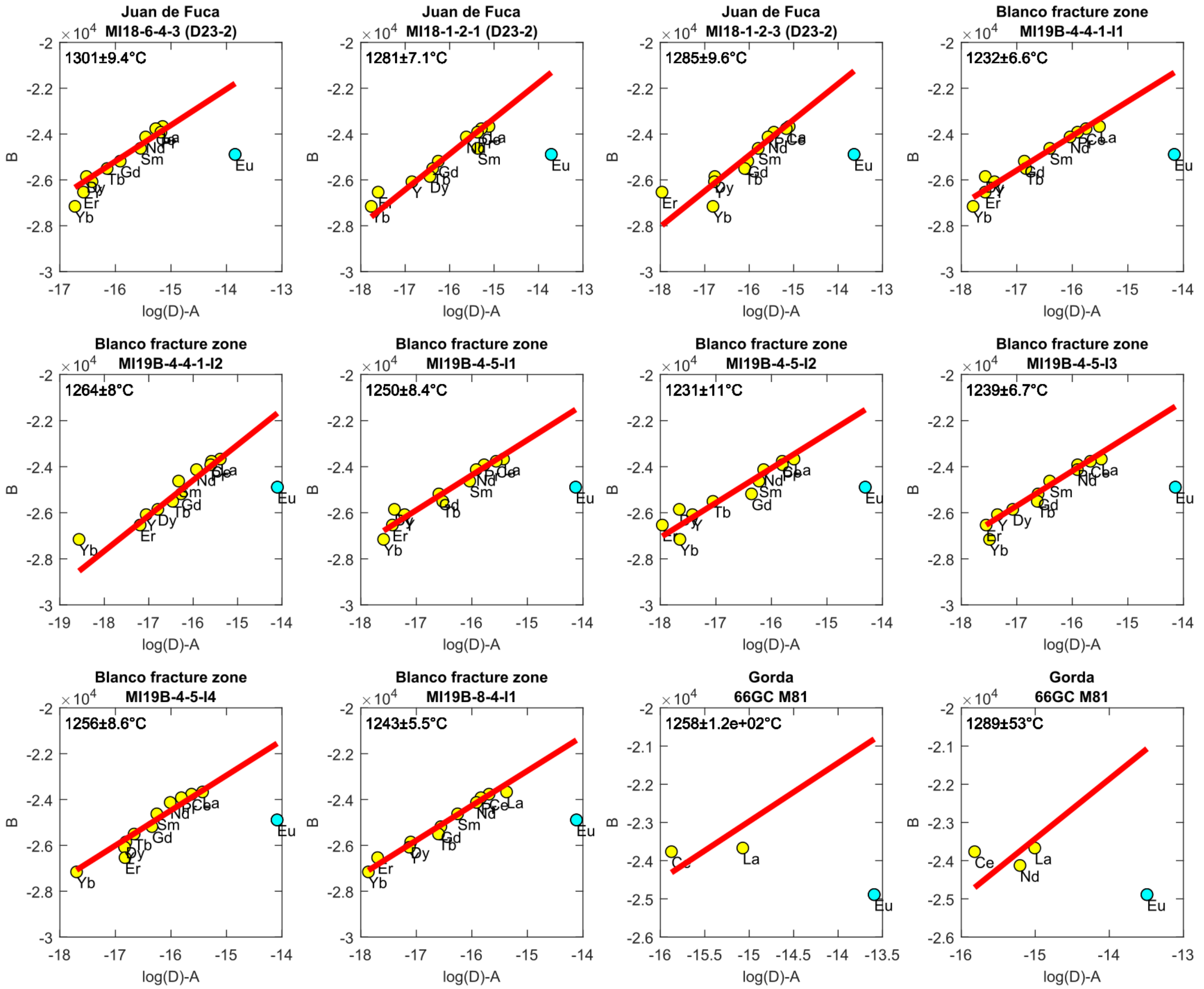

Fig. S5. Temperature inversions for samples investigated in this study and complied from the literature. Eu (flagged in light blue) falls off the trends defined by trivalent elements, and is excluded from the temperature determinations by a robust fitting algorithm. Uncertainty in the recovered temperatures is estimated using uncertainty in the slope of the best fit line. See Supplementary Data 3 for a summary of the input data and results.

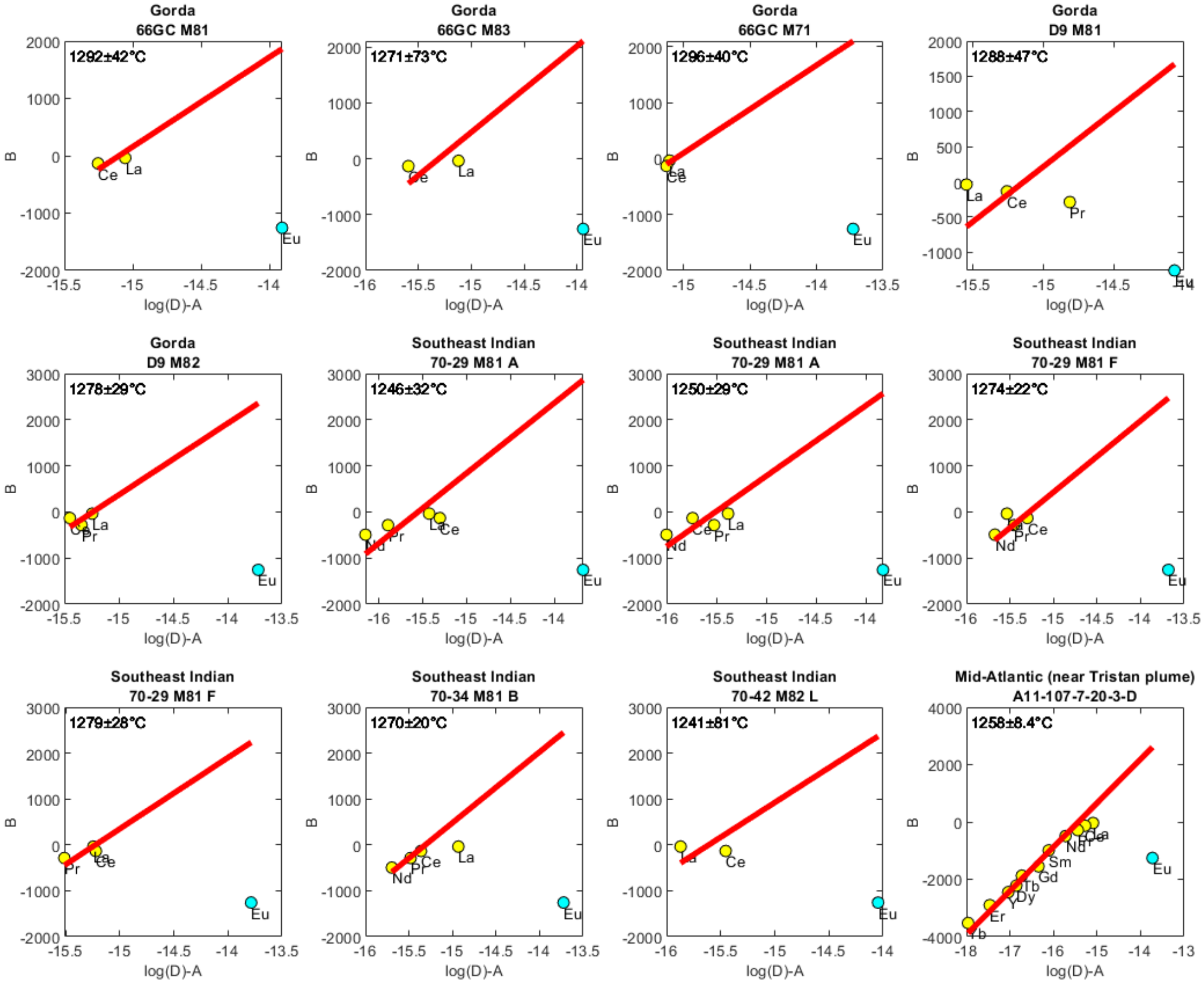

Fig. S6. Temperature inversions for samples investigated in this study and complied from the literature. Eu (flagged in light blue) falls off the trends defined by trivalent elements, and is excluded from the temperature determinations by a robust fitting algorithm. Uncertainty in the recovered temperatures is estimated using uncertainty in the slope of the best fit line. See Supplementary Data 3 for a summary of the input data and results.

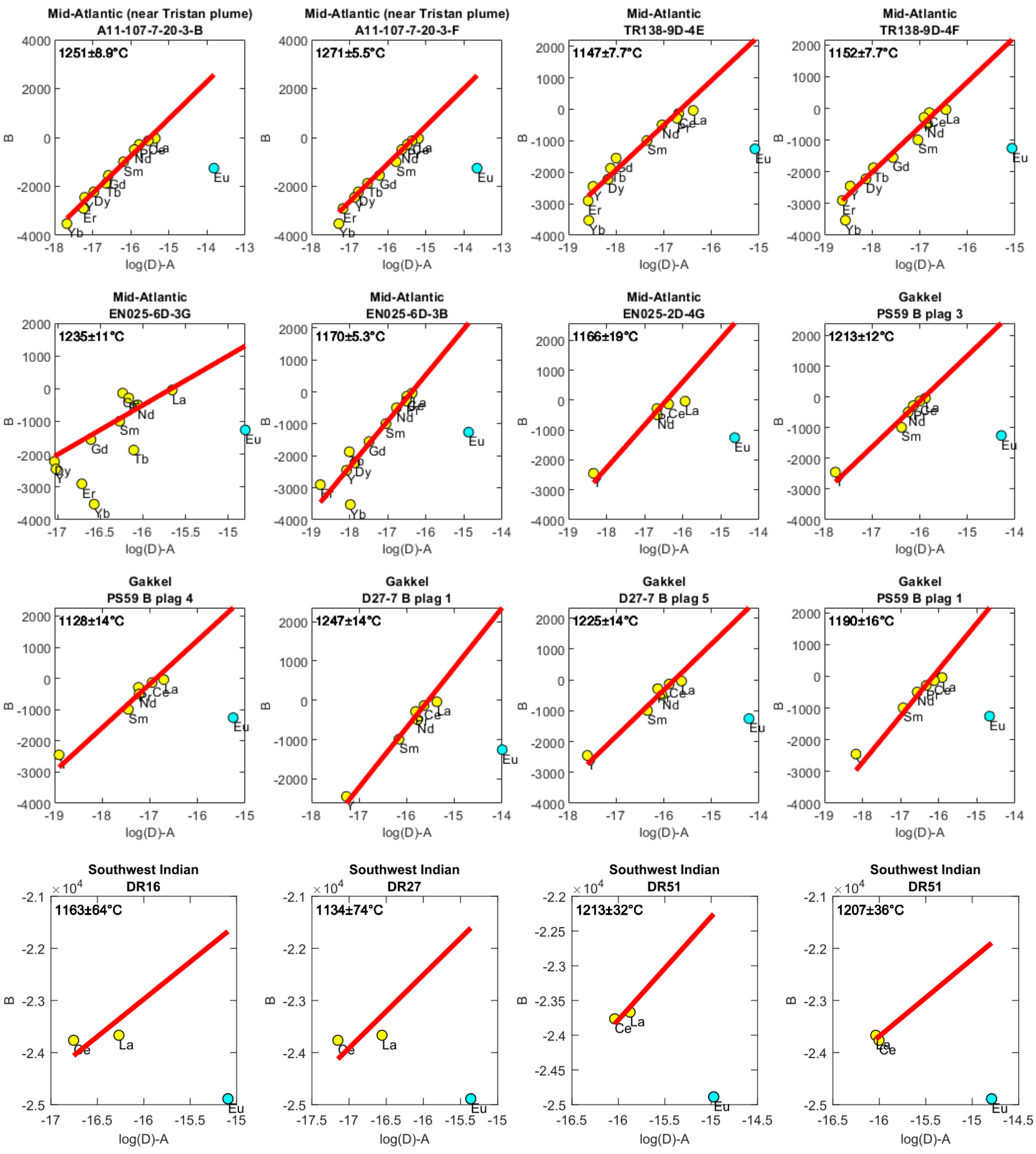

Fig. S7. Temperature inversions for samples investigated in this study and complied from the literature. Eu (flagged in light blue) falls off the trends defined by trivalent elements, and is excluded from the temperature determinations by a robust fitting algorithm. Uncertainty in the recovered temperatures is estimated using uncertainty in the slope of the best fit line. See Supplementary Data 3 for a summary of the input data and results.

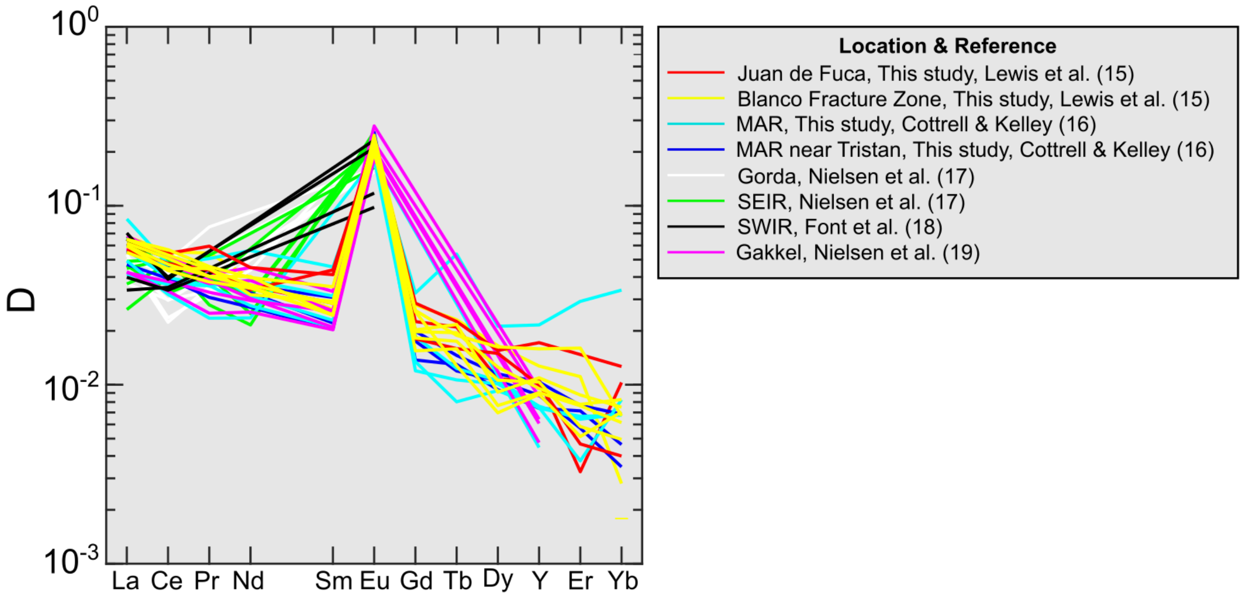

Fig. S8. Partition coefficients calculated using trace element data summarized in Supplementary Data 2 and 3.

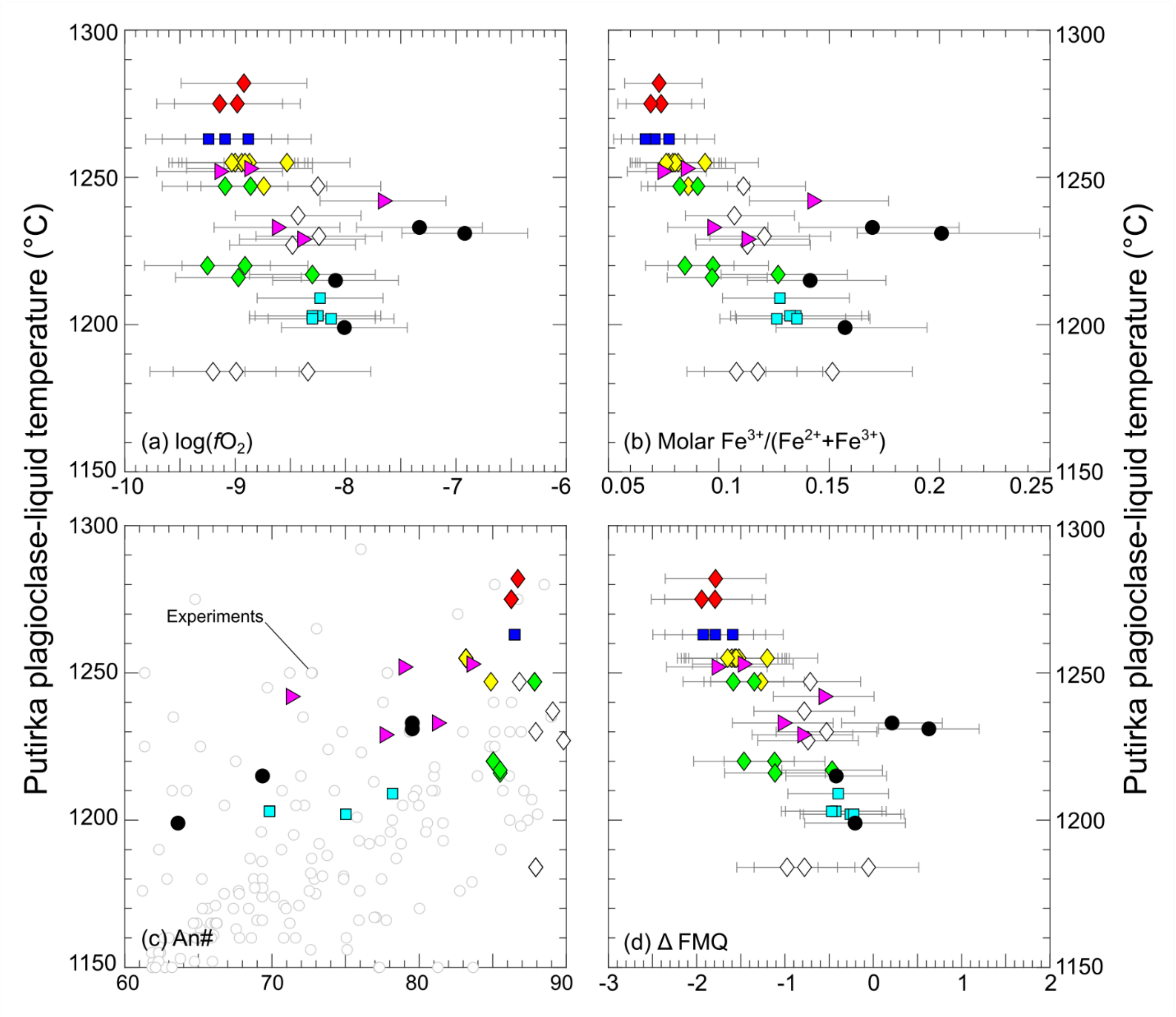

Fig. S9. Temperatures calculated using the plagioclase-liquid thermobarometer of Putirka[1] plotted against the log of the oxygen fugacity log(fO_2) recovered using Eqs. 7-15 (a), molar $Fe^{3+}/(Fe^{2+}+Fe^{3+})$ in glass calculated using the model of Kress and Carmichael[20] (b), plagioclase An# (100×Ca/(Ca+Na+K), in moles) (c), and relative to the fayalite-magnetite-quartz buffer (Δ FMQ) at 0.4 GPa[14] (d). Circles in the background of (c) are plagioclase-saturated experiments with oceanic basalt and basaltic andesite liquids (downloaded from the LEPR database[21]). Despite the more limited range of temperatures recovered using Putirka's thermometer than Eqs. 16-19, the variations of temperature with $Fe^{3+}/(Fe^{2+}+Fe^{3+})$ and Δ FMQ are qualitatively consistent with trends shown in Fig. 5 in the main text. Comparison of Fig. 5c and Supplementary Fig. S9c demonstrates that the REE equilibration temperature inversion method produces covariations of temperature and An# more consistent with experiments.

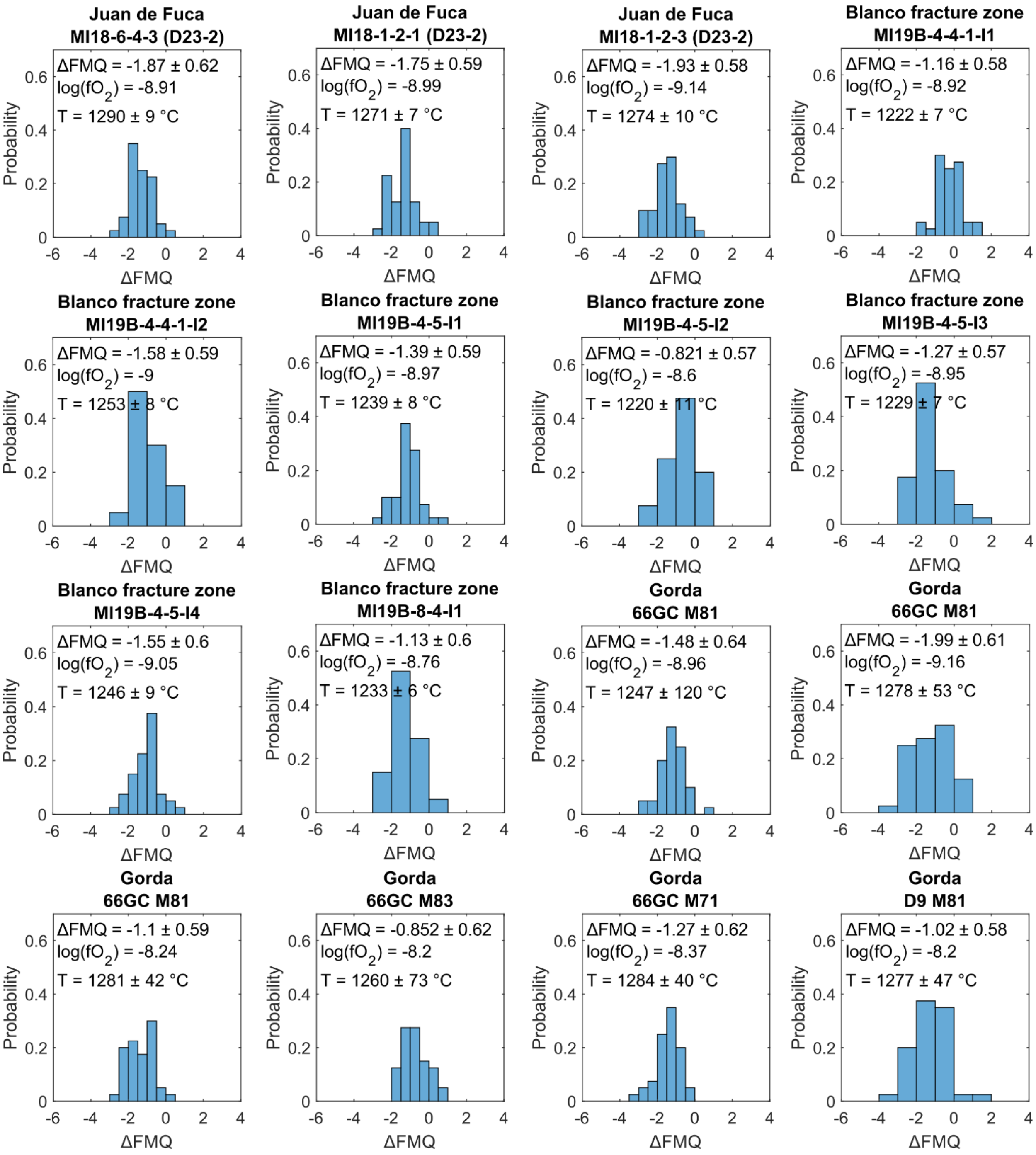

Fig. S10. Histograms showing distributions of oxygen fugacities (fO_2s) recovered using Eqs. 7-19 for the samples investigated in this study using Monte Carlo simulations. Deviation from the fayalite-magnetite-quartz buffer (ΔFMQ) is calculated using the parameterization of Frost[14]. Reported fO_2 uncertainty is the standard deviation of fO_2s calculated from 1000 synthetically perturbed datasets of input parameters. The simulations incorporate the following sources of uncertainty: P, T, K, major and trace element compositions, and lattice strain model coefficients (Eqs. 6a-7c, Sun et al., 2017[5]), see Methods for details.

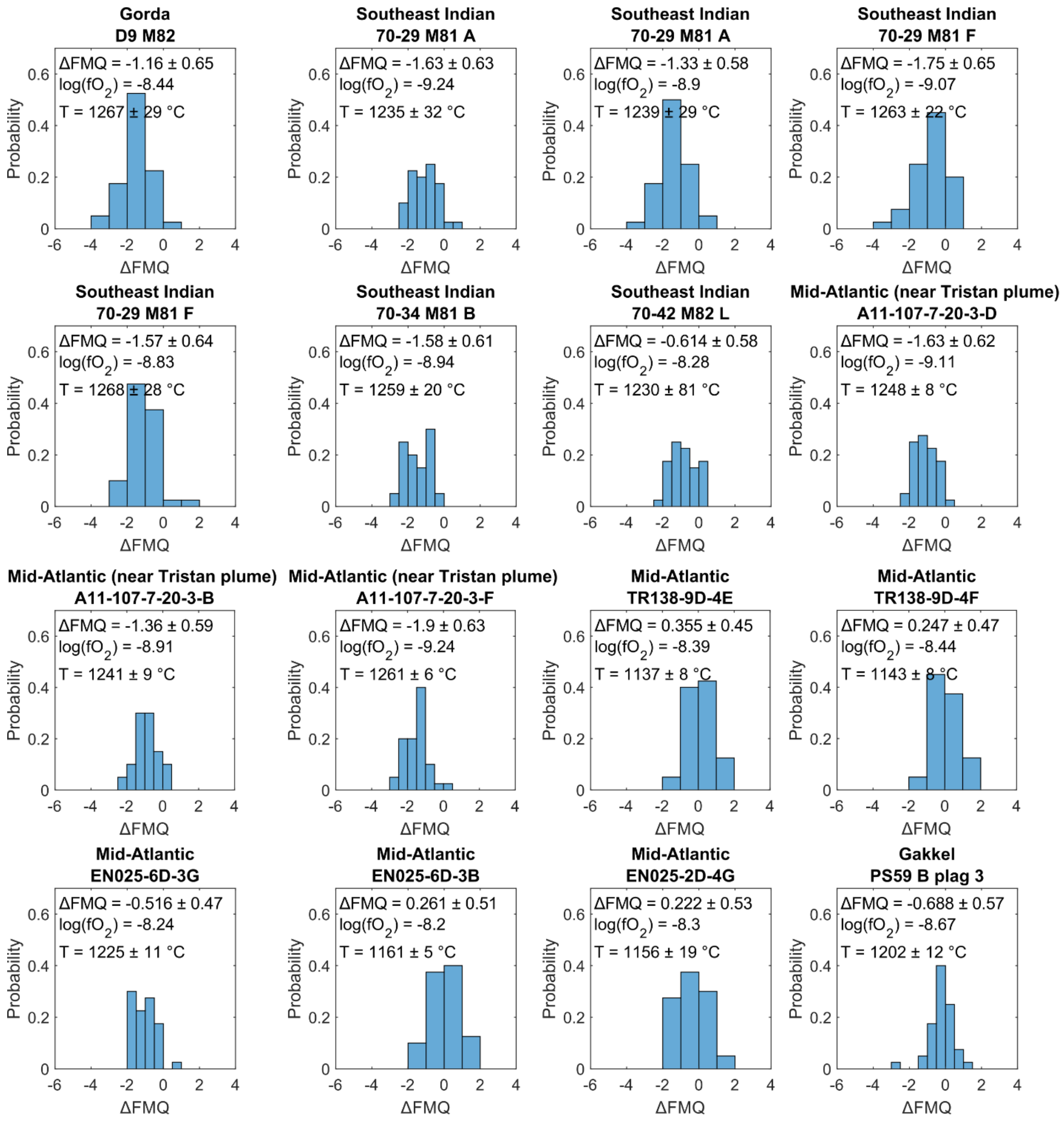

Fig. S11. Histograms showing distributions of oxygen fugacities (fO_2s) recovered using Eqs. 7-19 for the samples investigated in this study using Monte Carlo simulations. Deviation from the fayalite-magnetite-quartz buffer (ΔFMQ) is calculated using the parameterization of Frost[14]. Reported fO_2 uncertainty is the standard deviation of fO_2s calculated from 1000 synthetically perturbed datasets of input parameters. The simulations incorporate the following sources of uncertainty: P, T, K, major and trace element compositions, and lattice strain model coefficients (Eqs. 6a-7c, Sun et al., 2017[5]), see Methods for details.

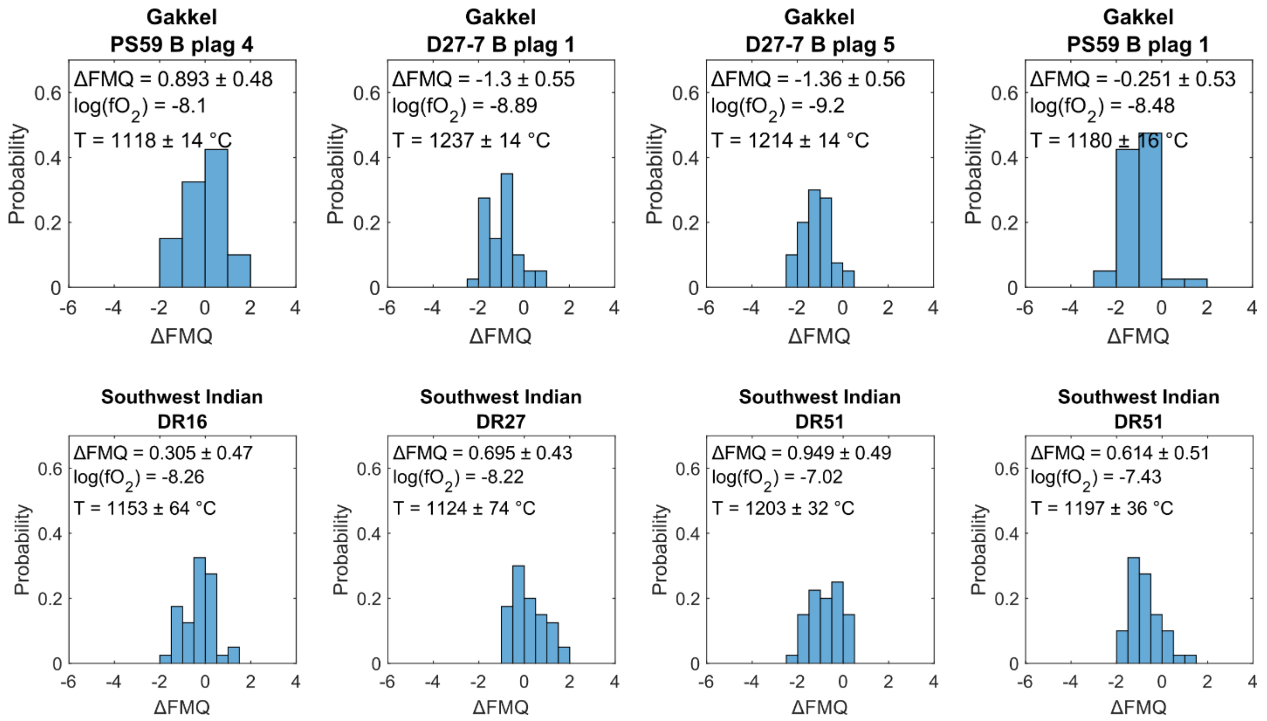

Fig. S12. Histograms showing distributions of oxygen fugacities (fO_2s) recovered using Eqs. 7-19 for the samples investigated in this study using Monte Carlo simulations. Deviation from the fayalite-magnetite-quartz buffer (ΔFMQ) is calculated using the parameterization of Frost[14]. Reported fO_2 uncertainty is the standard deviation of fO_2s calculated from 1000 synthetically perturbed datasets of input parameters. The simulations incorporate the following sources of uncertainty: P, T, K, major and trace element compositions, and lattice strain model coefficients (Eqs. 6a-7c, Sun et al., 2017[5]), see Methods for details.

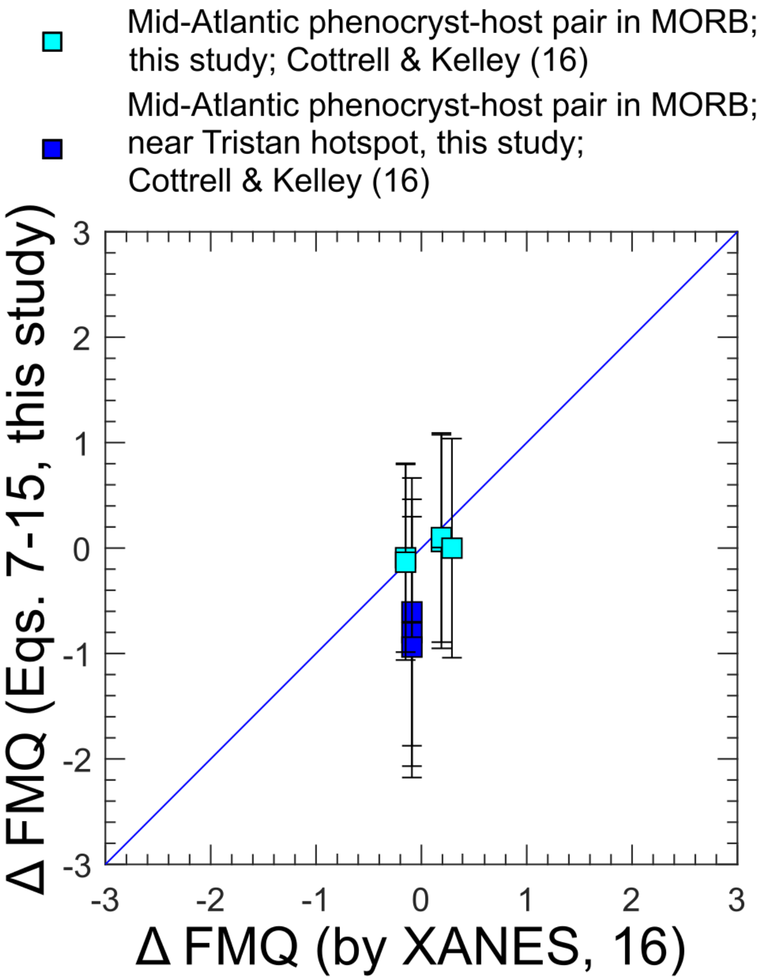

Fig. S13. A comparison of oxygen fugacities (fO_2s) relative to the fayalite-magnetite-quartz buffer[14] (Δ FMQ) at atmospheric pressure and 1200°C calculated using measured Eu distributions and major element compositions (Eqs. 7-15, this study, y axis) and an Fe-speciation based method, X-ray absorption near edge spectroscopy (x axis, Cottrell and Kelley[16]). Uncertainties in the Eu-based method are propagated through the oxybarometer as described in the Methods, and as shown in Supplementary Fig. S11. The error bars in this figure are plotted as 2σ uncertainties, data in the main text are plotted with 1σ uncertainties. The comparison demonstrates that the Eu-in-plagioclase-melt method (Eqs. 7-15) recovers fO_2s consistent with XANES-based methods from natural samples.

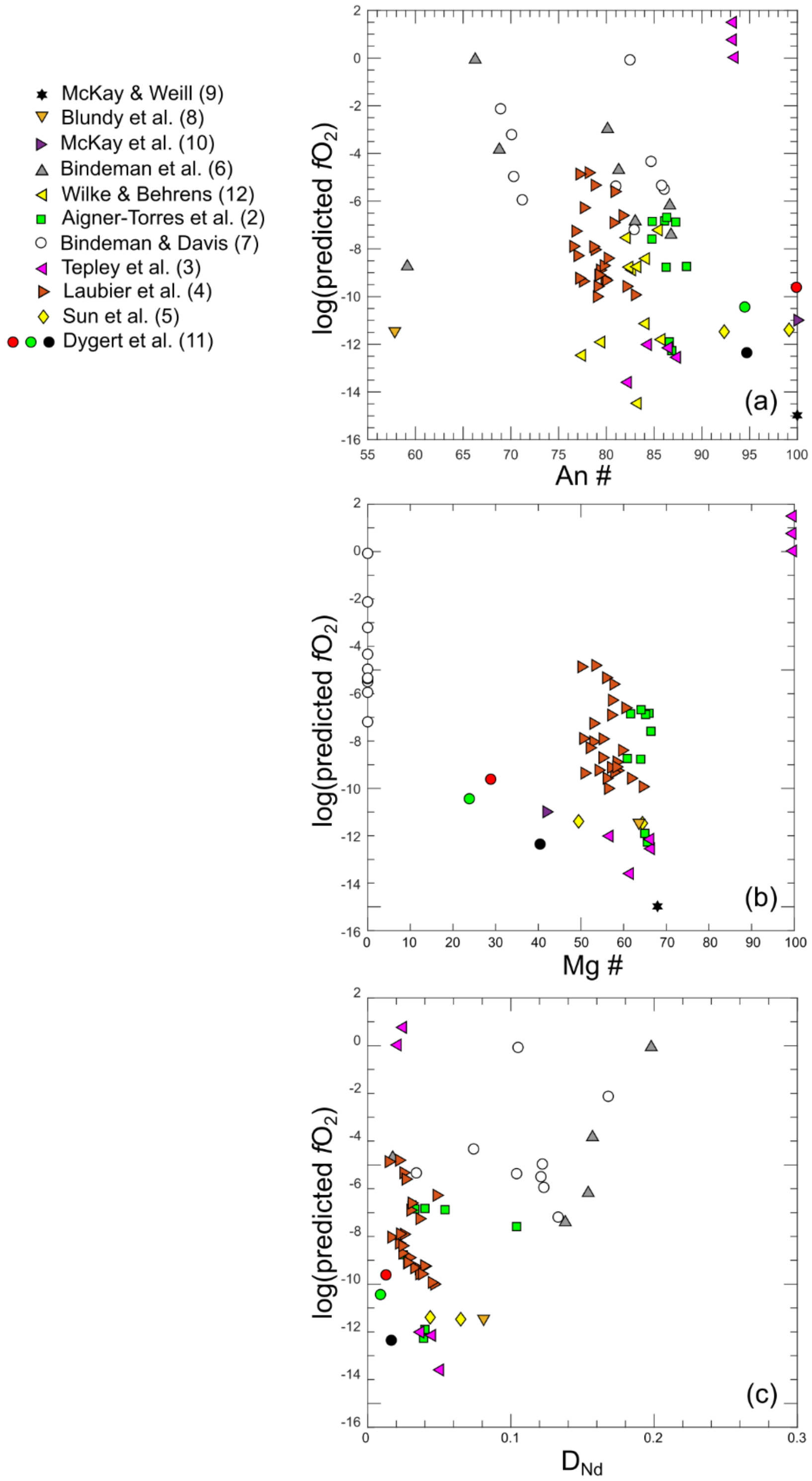

Fig. S14. Oxygen fugacities (fO_2s) calculated from application of Eqs. 7-15 to the experimental compilation of Dygert et al.[11], plotted against plagioclase An# (100×Ca/(Ca+Na), in moles) (a), melt Mg# (100×Mg/(Mg+Fe), in moles) (b), and Nd partition coefficient (c; D_{Nd}). There is no systematic covariation of fO_2 with An#, melt Mg#, or D_{Nd}.

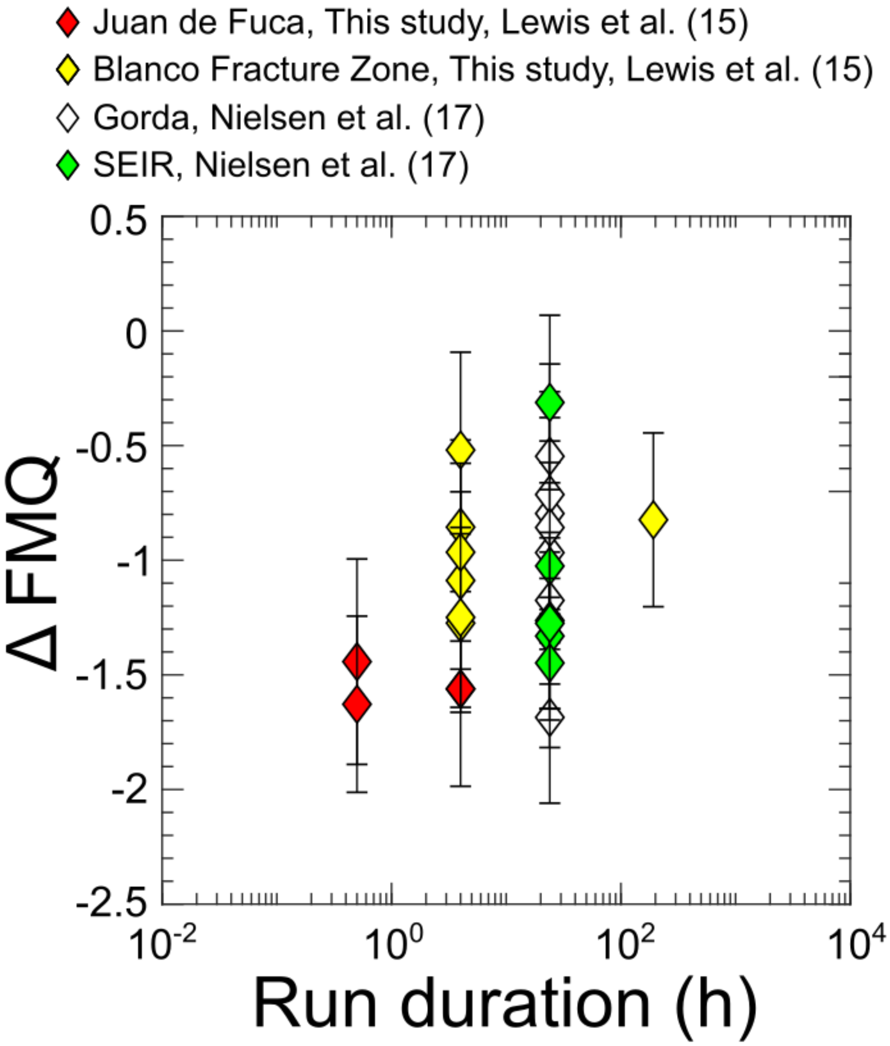

Fig. S15. Oxygen fugacities (fO_2s) determined using measured plagioclase-melt inclusion glass Eu distributions relative to the fayalite-magnetite-quartz buffer[22] (Δ FMQ) at atmospheric pressure plotted against experimental homogenization time (hours). Melt inclusion-bearing phenocrysts are heated to their estimated liquidus temperatures in air in a vertical tube furnace, then quenched (see the Methods for details). Note the fO_2s are consistent with origination of the melt inclusions from relatively reduced sources, and do not exhibit systematic correlation of recovered fO_2 with experimental time, consistent with them being unperturbed by the homogenization procedure.

References

1. Putirka, K. D. Thermometers and barometers for volcanic systems. *RiMG* **69**, 61-120 (2008).
2. Aigner-Torres, M., Blundy, J., Ulmer, P. & Pettke, T. Laser Ablation ICPMS study of trace element partitioning between plagioclase and basaltic melts: an experimental approach. *Contrib. Mineral. Petrol.* **153**, 647-667 (2007).
3. Tepley, F. J., Lundstrom C. C., McDonough W. F. & Thompson A. Trace element partitioning between high-An plagioclase and basaltic to basaltic andesite melt at 1 atmosphere pressure. *Lithos* **118**, 82-94 (2010).
4. Laubier, M., Grove, T. L. & Langmuir, C. H. Trace element mineral/melt partitioning for basaltic and basaltic andesitic melts: An experimental and laser ICP-MS study with application to the oxidation state of mantle source regions. *Earth Planet. Sci. Lett.* **392**, 265-278 (2014).
5. Sun, C., Graff, M. & Liang, Y. Trace element partitioning between plagioclase and silicate melt: The importance of temperature and plagioclase composition, with implications for terrestrial and lunar magmatism. *Geochim. Cosmochim. Acta* **206**, 273-295 (2017).
6. Bindeman I., Davis A., & Drake M. J. Ion microprobe study of plagioclase-basalt partition experiments at natural concentration levels of trace elements. *Geochim. Cosmochim. Acta* **62**, 1175–1193 (1998).
7. Bindeman I. & Davis, A. Trace element partitioning between plagioclase and melt: investigation of dopant influence on partition behavior. *Geochim. Cosmochim. Acta* **64**, 2863-2878 (2000).
8. Blundy J. D., Robinson J. A. C. & Wood B. J. Heavy REE are compatible in clinopyroxene on the spinel lherzolite solidus. *Earth Planet. Sci. Lett.* **160**, 493-504, (1998).
9. McKay, G. & Weill, D. F. KREEP petrogenesis revisited. *Proc. 8th Lunar Plan Sci Conf.* **2**, 2339-2355 (1977).
10. McKay G., Le, L., Wagstaff, J. & Crozaz, G. Experimental partitioning of rare earth elements and strontium: Constraints on petrogenesis and redox conditions during crystallization of Antarctic angrite Lewis Cliff 86010. *Geochim. Cosmocim. Acta* **58**, 2911-2919 (1994).
11. Dygert, N., Draper, D. S., Rapp, J. F., Lapen, T. J., Fagan, A. L. & Neal, C. R. Experimental determinations of trace element partitioning between plagioclase, pigeonite, olivine, and lunar basaltic melts and an fO2 dependent model for plagioclase-melt Eu partitioning. *Geochim. Cosmochim. Acta* **279**, 258-280 (2020).
12. Wilke M. & Behrens H. The dependence of the partitioning of iron and europium between plagioclase and hydrous tonalitic melt on oxygen fugacity. *Contrib. Mineral. Petrol*. **137**, 102-114 (1999).
13. O'Neill, H.S.C. & Pownceby, M.I. Thermodynamic data from redox reactions at high temperatures. I. An experimental and theoretical assessment of the electrochemical method using stabilized zirconia electrolytes, with revised values for the Fe-"FeO", Co-CoO, Ni-NiO and Cu-Cu_2O oxygen buffers, and new data for the W-WO_2 buffer. *Contrib. Mineral. Petrol.* **114**, 296-614 (1993).
14. Frost, B. R. Introduction to oxygen fugacity and its petrologic importance. *Rev. Mineral. Geochem.* **25**, 1-9 (1991).

15. Lewis, K. R., Ustunisik, G. K. & Nielsen, R. L. Experimental Constraints on Homogenization of Plagioclase-Hosted Melt Inclusions from Plagioclase Ultraphyric Basalts. *Front. Earth Sci*. **8**, doi:10.3389/feart.2020.584371 (2021).
16. Cottrell, E. & Kelley, K. A. The oxidation state of Fe in MORB glasses and the oxygen fugacity of the upper mantle. *Earth Planet. Sci. Lett*. **305**, 270-282 (2011).
17. Nielsen, R. L., Ustunisik, G., Weinsteiger, A. B., Tepley, F. J., Johnston, A. D. & Kent, A. J. R. Trace element partitioning between plagioclase and melt: An investigation of the impact of experimental and analytical procedures. *Geochem. Geophys. Geosyst.* **18**, doi:10.1002/2017GC007080 (2017).
18. Font, L., Murton, B. J., Roberts, S. & Tindle, A. G. Variations in Melt Productivity and Melting Conditions along SWIR (70°E-49°E): Evidence from Olivine-hosted and Plagioclase-hosted Melt Inclusions. *J. Pet.* **48**, 1471-1494 (2007).
19. Nielsen, R. L., Ustunisik, G., Lange, A. E., Tepley, F. J. & Kent, A. J. R. Trace Element and Isotopic Characteristics of Plagioclase Megacrysts in Plagioclase Ultraphyric Basalts (PUB). *Geochem. Geophys. Geosyst*. **21**, doi:10.1029/2019GC008638 (2020).
20. Kress, V. C. & Carmichael, I. S. E. The compressibility of silicate liquids containing Fe_2O_3 and the effect of composition, temperature, oxygen fugacity and pressure on their redox states. *Contrib. Mineral. Petrol*. **108**, 82-92 (1991).
21. Hirschmann, M.M., Ghiorso, M.S., Davis, F.A., Gordon, S.M., Mukerjee, S., Grove, T.L., Krawczynski, M., Medard, E. & Till, C.B. Library of Experimental Phase Relations (LEPR): A database and web portal for experimental magmatic phase equilibria. *Geochem. Geophys. Geosyst.* **9**, Q03011 (2008).
22. Myers, J. & Eugster, H.P. The system Fe-Si-O: Oxygen Buffer Calibrations to 1,500K. *Contrib. Mineral. Petrol.* **82**, 75-90 (1998).
