# Supplementary material for: Europium in plagioclase-hosted melt inclusions reveals mantle melting modulates oxygen fugacity
Source: Nat Commun. 2024 Apr 8;15:3033. doi: 10.1038/s41467-024-47224-5 (PMC11001916; doi:10.1038/s41467-024-47224-5)
Supplement: Supplementary file 2 — Description of Additional Supplementary Files [file 41467_2024_47224_MOESM2_ESM.pdf]

Description of Additional Supplementary Files

Supplementary Data Legends

Supplementary Data 1: Major element analyses.

Supplementary Data 2: Trace element analyses.

Supplementary Data 3: Compilation of new data produced in this study and data from the literature, and calculated oxygen fugacities.

Supplementary Data 4: Major element analyses of plagioclase around melt inclusions in crystals experimentally homogenized for different periods of time, from sample D23-2, Juan de Fuca Ridge.

Supplementary Data 5: Secondary standard recoveries from laser ablation inductively coupled plasma mass spectrometry analyses.

Supplementary Software 1: A REE-in-Plagioclase-Melt Thermometer

A thermometer for recovering temperatures from distributions of REEs between plagioclase and coexisting glasses.
